# PTEN suppresses tumorigenesis by directly dephosphorylating Akt

**DOI:** 10.1038/s41392-021-00571-x

**Published:** 2021-07-12

**Authors:** Lang Bu, Huan Wang, Ji-an Pan, Lang Chen, Fan Xing, Junyu Wu, Shun Li, Deyin Guo

**Affiliations:** 1 Institute of Precision Medicine, the First Affiliated Hospital, School of Medicine, Sun Yat-sen University, Guangzhou, Guangdong China; 2 Department of Immunology, School of Basic Medical Sciences, Wuhan University, Wuhan, Hubei China; 3 Modern Virology Research Center, College of Life Sciences, Wuhan University, Wuhan, Hubei China; 4 Present Address: Institute of Synthetic Biology, Institutes of Advanced Technologies, Shenzhen, Guangdong China

**Keywords:** Molecular biology, Oncogenes

**Dear Editor**,

The serine-threonine kinase Akt plays a central role in regulating cell proliferation, migration, angiogenesis, transformation, energy metabolism, and death.^1^ The stimulation of growth factors recruits the PI3K to the plasma membrane and phosphatidylinositol-3,4,5-trisphosphate (PIP3), which in turn recruits Akt to the plasma membrane through PH domain of Akt. Then Akt is phosphorylated at T308 by PDK1 and at S473 by mammalian target of rapamycin complex 2 (mTORC2).^2^ PTEN is one of the most important tumor suppressors, which possesses both lipid and protein phosphatase activity. PTEN exerts its tumor suppression function through the dephosphorylation of PIP3 and antagonizing PI3K-Akt signaling pathway with its lipid phosphatase activity.^3,4^ The previous study also suggests that the lipid phosphatase activity of PTEN does not play a role in suppression of Akt in the nucleus.^5,6^ New evidence indicates that PTEN may negatively regulate the activation of Akt independently of its lipid phosphatase activity, but the molecular details are still obscure.

By genome-wide bioinformatics analysis, we searched the proteins that contain the conserved phosphorylation motif (R/KXR/KXX*S/T; X, any amino acid) recognized by Akt. PTEN contains the motif which is well conserved in different species (Fig. 1a). We confirmed the interaction among PTEN and Akt, and the signals of PTEN and Akt1 overlapped remarkably in the cells (Fig. 1b–d and Supplementary Fig. S1a–d and S1g–j, and Supplementary video). PTEN interacted with Akt1 through its C2 domain and Akt1 interacted with PTEN through the central kinase domain (Supplementary Fig. S1e, f). Furthermore, PTEN interacted with the other two Akt family members: Akt2 and Akt3 (Supplementary Figs. S1k–m and S2a–i). Our results conclude that PTEN directly interacts with Akt1, 2, and 3. In addition, the S226 site of PTEN could be phosphorylated by Akt1 and the phosphorylation of this site could promote the interaction between PTEN and Akt (Supplementary Fig. S3a–d).

**Fig. 1:**
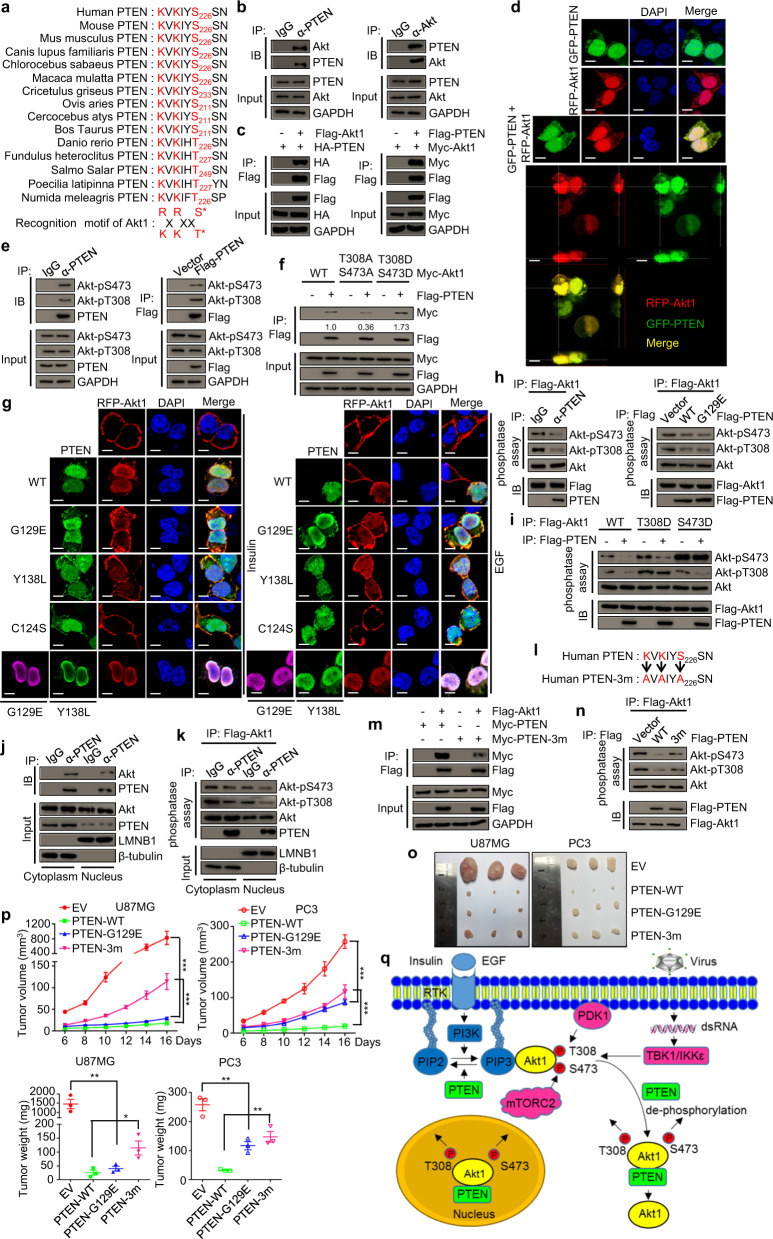
PTEN suppresses tumorigenesis by directly dephosphorylating Akt. **a** Sequence comparison of PTEN gene in different species. The phosphorylation motifs of Akt are highlighted in red. **b** Whole-cell lysates of 293T cells were collected, immunoprecipitated with indicated antibodies (IP) and subjected to immunoblotting (IB) analysis. **c** 293T cells were transfected with vector, Flag-Akt1, or Flag-PTEN. The cell lysates were prepared for IP and IB analysis. **d** 293T cells were transfected with GFP-PTEN and RFP-Akt1 separately or together. IF assays were performed. Scale bar is 10 μm. **e** 293T cells were collected or transfected with vector or Flag-PTEN, then cells were subjected to IP and IB analysis. **f** 293T cells were co-transfected with vector or Flag-PTEN and Myc-Akt1, Myc-Akt1-T308A-S473A, or Myc-Akt1-T308D-S473D. Cells were subjected to IP and IB analysis. **g** 293T cells were transfected with RFP-Akt1 alone or together with Flag-PTEN, Flag-PTEN-G129E, Flag-PTEN-Y138L, or Flag-PTEN-C124S respectively, or co-transfected with RFP-Akt1, Flag-PTEN-G129E, and Myc-PTEN-Y138L. The cells were serum-starved for 18 h, and then treated with insulin (1 μg/ml) or EGF (100 ng/ml) for 10 min before being subjected to IF assays. Scale bar, 10 μm. **h** 293T cells were transfected with vector, Flag-PTEN, Flag-Akt1, Flag-PTEN-G129E respectively, immunoprecipitated with anti-Flag agarose, and eluted with 3× Flag peptide. Endogenous PTEN was immunoprecipitated with PTEN antibody from 293T cells. The purified proteins were subjected to phosphatase assays. **i** 293T cells were transfected with vector, Flag-PTEN, Flag-Akt1, Flag-Akt1-T308D, or Flag-Akt1-S473D respectively, immunoprecipitated with anti-Flag agarose, and eluted with 3× Flag peptide. The purified proteins were subjected to phosphatase assays. **j** The nuclear and cytoplasmic fraction of 293T cells were extracted and immunoprecipitated with PTEN antibody. The IP samples were subjected to IB analysis. **k** Flag-Akt1 was transfected into 293T cells. The whole cell extract was immunoprecipitated with anti-Flag agarose and eluted with 3× Flag peptide. Endogenous PTEN was purified from nuclear and cytoplasmic extracts of 293T cells with immunoprecipitation. Flag-Akt1 and PTEN proteins were subjected to phosphatase assays and reaction samples were examined by IB analysis. **l** The schematic for PTEN-3m (PTEN-K221A-K223A-S226A mutant). **m** 293T cells were co-transfected with Myc-PTEN and vector or Flag-Akt1, or co-transfected with Myc-PTEN-3m and vector or Flag-Akt1. The cells were subjected to IP and IB analysis. **n** 293T cells were transfected with vector, Flag-PTEN, Flag-PTEN-3m, or Flag-Akt1 respectively. The cells were immunoprecipitated with anti-Flag agarose and eluted with 3× Flag peptide. The purified proteins were subjected to phosphatase assays. **o** PTEN-depleted U87MG cells or PC3 cells stably expressing vector, PTEN, PTEN-G129E, or PTEN-3m were subcutaneously injected into nude mice (*n* = 3 per group) as described in the Methods section. The formed tumors as indicated were shown. **p** The dissected tumor weights were measured, and tumor volumes were calculated. Data were represented as means ± SEM. *P < 0.05; **P < 0.01; ***P < 0.001, based on the Student’s *t* tests or two-way ANOVA. **q** A model of how PTEN suppresses the activity of Akt. The experiments in b, d–g, and i–k were performed two times independently with similar results. The experiments in c, h, and m-n were performed three times independently with similar results.

As the activation of Akt is strictly regulated by the upstream signals, we interrogated the regulatory effect of growth factors or viral infection on the interaction between Akt and PTEN. The interaction between Akt1 and PTEN were increased upon the stimulation of insulin, EGF, or SeV (Supplementary Fig. S4a–g). Then we investigated the molecular details which account for the enhanced the interaction. We confirmed that PTEN interacted with phosphorylated Akt1 (pS473 and pT308) (Fig. 1e and Supplementary Fig. S1i). And more phosphorylated Akt1 mimics were detected in PTEN-associated protein precipitates, and mutations (Akt1-T308A-S473A) that impaired the phosphorylation of Akt1 largely attenuated the interaction between Akt1 and PTEN (Fig. 1f). Taken together, our data indicate that PTEN prefers to bind to phosphorylated Akt1.

Given the finding that PTEN directly interacts with Akt and this interaction is associated with the phosphorylation of Akt, we asked whether the protein phosphatase activity of PTEN plays a role in the recruitment of Akt to the plasma membrane, which is important for the phosphorylation of Akt. PTEN or co-expression of PTEN-G129E (lipid phosphatase-deficient mutant), and PTEN-Y138L (protein phosphatase-deficient mutant) could strongly block the recruitment of Akt1 to the plasma membrane after stimulation by insulin or EGF, and that PTEN-G129E or PTEN-Y138L alone, but not PTEN-C124S (lipid and protein phosphatase-deficient mutant), could partially inhibit the recruitment of Akt1 (Fig. 1g and Supplementary Fig. S5a–d). PTEN also strongly blocked the recruitment of Akt2 and Akt3 to the plasma membrane after the stimulation (Supplementary Fig. S5e, f). Collectively, PTEN can inhibit the stimulation-induced recruitment of Akt1 to the plasma membrane in a lipid phosphatase- and protein phosphatase-dependent manner.

As aforementioned, lipid phosphatase-deficient PTEN-G129E can inhibit the membrane recruitment of Akt. To rule out the influence of the lipid phosphatase activity, we inhibited the activity of PI3K with pharmacological inhibitors or constructed PI3K knockout (PIK3CA^−/−^ PIK3CB^−/−^ PIK3R1^−/−^) 293T cell line. Under the condition that PTEN could not exert its lipid phosphatase function, it still inhibited the phosphorylation of Akt1 (Supplementary Figs. S6a–e and S7a–c). PTEN is a general regulator of Akt signaling and its regulatory effect is not limited lipid phosphatase activity.

Next, we observed that PTEN and PTEN-G129E could dephosphorylate Akt1 directly in vitro, while PTEN-Y138L and PTEN-C124S could not (Fig. 1h and Supplementary Fig. S8a–f). To investigate the order of dephosphorylations on the two sites of Akt1, T308 and S473, we checked whether PTEN could dephosphorylate the Akt1-T308D or Akt1-S473D. The results showed that Akt1-T308D and Akt1-S473D could affect the dephosphorylation effect of PTEN on p-T308 and p-S473 respectively, indicating that the dephosphorylations on two sites happen independently (Fig. 1i). Notably, we found that the protein phosphatase activity of PTEN could not inhibit the recruitment of Akt1-T308A-S473A and Akt1-T308D-S473D to the plasma membrane after stimulating by insulin (Supplementary Fig. S8g, h). Taken together, PTEN can directly dephosphorylate Akt1 at S473 and T308 by its protein phosphatase activity.

Then we asked whether PTEN dephosphorylates Akt modified by different known Akt kinases. PTEN inhibited the phosphorylation of Akt1 induced by classical or non-classical pathways through its protein phosphatase activity (Supplementary Figs. S7d, e and S9a–i). In order to avoid the inhibitory effect of PIP3 by PTEN on cell membrane, we generated a series of PTEN mutants with different subcellular localization signals: PTEN with nuclear localization signal (NLS-PTEN), PTEN with ER localization signal (ER-PTEN), PTEN with mitochondrial localization signal (Mito-PTEN), and PTEN with plasma membrane localization signal (Myr-PTEN). The different subcellular localizations of PTEN without effect on PIP3 interacted with Akt1 and decreased the phosphorylation of Akt1 (Supplementary Fig. S10a–h). The previous study also suggests that the lipid phosphatase activity of PTEN does not play a role in suppression of Akt in the nucleus, and we observed that nuclear PTEN dephosphorylated nuclear Akt1 (Fig. 1j, k and Supplementary Fig. S11a–f). Our results depict the molecular mechanism that nuclear PTEN inhibits the activity of nuclear Akt through the direct dephosphorylation.

Next, we investigated the role of interaction between Akt1 and PTEN in the dephosphorylation of Akt1. We confirmed that the mutation of conserved residues K221, K223, and S226 to alanine (PTEN-3m) drastically decreased the interaction between Akt1 and PTEN, and the dephosphorylation of Akt1 by PTEN is also attenuated (Fig. 1l–n and Supplementary Fig. S12a–d). To test whether PTEN can suppress tumorigenesis by targeting Akt via its protein phosphatase activity, we adopted tumor formation assays with two PTEN-deficient cell lines, prostate cancer cells PC3 and glioma-derived cells U87MG. PTEN (wild-type), PTEN-G129E (which is deficient in lipid phosphatase activity), and PTEN-3m (which has lower protein phosphatase activity on Akt1) could inhibit the tumorigenesis, whereas the PTEN-3m had a less inhibitory effect on tumor formation than PTEN (Fig. 1o, p and Supplementary Fig. S13a–g).

Here, we find that PTEN interacts with Akt and dephosphorylates Akt1 at S473 and T308 sites, thus negatively regulates Akt1 activity by short-circuiting the classical PTEN-PIP3-PDK1-Akt signaling pathway. The S226 site of PTEN can be phosphorylated by Akt1. PTEN inhibits growth factor-induced recruitment of Akt1 to the plasma membrane in a lipid phosphatase- and protein phosphatase-dependent manner. Given that PTEN recognizes the phosphorylated Akt1, PTEN can also interact with and dephosphorylate Akt1 in different cellular compartments, including the nucleus (Fig. 1q). PTEN variant with protein phosphatase activity but deficient in lipid phosphatase activity can suppress tumorigenesis in a mouse model, suggesting a physiological role of PTEN protein phosphatase activity in the suppression of tumorigenesis. Our findings represent a conceptual advance in the function and mechanism of PTEN in regulation of Akt activity and in suppression of tumorigenesis.

## Supplementary information

Supplementary material

Supplementary Figures

Supplementary video
