# Supplementary material for: PTEN suppresses tumorigenesis by directly dephosphorylating Akt
Source: Signal Transduct Target Ther. 2021 Jul 12;6:262. doi: 10.1038/s41392-021-00571-x (PMC8273154; doi:10.1038/s41392-021-00571-x)
Supplement: Supplementary file 1 — Supplementary material [file 41392_2021_571_MOESM1_ESM.docx]

Supplementary Materials for

**PTEN suppresses tumorigenesis by directly dephosphorylating Akt**

Lang Bu^1#^, Huan Wang^1#^, Ji-an Pan^1^, Lang Chen^2^, Fan Xing^1^, Junyu Wu^1^, Shun Li^3,†^, Deyin Guo^1*^

^1^ Institute of Precision Medicine, the First Affiliated Hospital, School of Medicine, Sun Yat-sen University, Guangzhou 510080, Guangdong, China;

^2^ Department of Immunology, School of Basic Medical Sciences, Wuhan University, Wuhan 430071, Hubei, China;

^3^ Modern Virology Research Center, College of Life Sciences, Wuhan University, Wuhan 430071, Hubei, China;

#These authors contributed equally: Lang Bu, Huan Wang

†Current address: Institute of Synthetic Biology, Institutes of Advanced Technologies, Shenzhen, China

*Correspondence: Deyin Guo, School of Medicine, Sun Yat-sen University, Guangzhou 510080, Guangdong, China. E-mail: guodeyin@mail.sysu.edu.cn

**This file includes:**

Materials and Methods

References for supplementary materials

Figures. S1 to S13

**Materials and Methods**

**Cell culture and virus**

*Pten^+/+^* MEFs, *Pten^-/-^* MEFs, WT MEFs, 293T, and PC3 cells have been described previously.^1^ PTEN-depleted U87MG cell line was a gift from Professor Guangmei Yan (Sun Yat-sen University). Akt1^-/-^ 293T cells and DLD1-Akt1/2^-/-^ cells were gifts from Professor Jianping Guo (Sun Yat-sen University). *Pten^+/+^* MEFs, *Pten^-/-^* MEFs, WT MEFs, 293T, and U87MG cells were cultured in DMEM medium (Hyclone) containing 10% FBS (Gibco). PC3 cells were cultured in RPMI medium (Hyclone) containing 10% FBS. All cell lines were tested for mycoplasma contamination and the results were negative. Cells were transfected using Lipofectamine 2000 (Invitrogen) according to the manufacturer’s instructions. Lentivirus packaging and subsequent infection of various cell lines were performed according to the protocol as previously described.^1^ SeV has been described previously.^1^

**Plasmids**

pEF-Flag-PTEN, pEF-Myc-PTEN, pEF-Flag-PTEN-C124S, pEF-Myc-PTEN-C124S, pEF-Flag-PTEN-G129E, pRK-HA-PTEN, pEGFP-C1-PTEN, pEF-Flag-TBK1, pEF-Flag-IKKε, pLKO.1-shPTEN, PHAGE-PTEN have described previously.^1^ pDNA3-CFP (#13030), pcDNA3-YFP (#13033), and pCMV-ECFP/EYFP (#24520) were bought from Addgene. Flag-mTOR and Flag-Rictor were gifts from Professor X.D. Xie (Sun Yat-sen University). pEF-Flag-NLS-PTEN, pEF-Flag-Mito-PTEN, and pEF-Flag-ER-PTEN were constructed according to life technologies (catalog number : V821-20, V822-20, V823-20) standard procedures. pEF-Flag-Myr-PTEN and pEF-Flag-Myr-Akt1 were constructed with the myristoylation sequence (MGSSKSKPK). pEF-Flag-Akt1, pEF-Myc-Akt1, pEF-Flag-Akt2, pEF-Myc-Akt2, pEF-Flag-Akt3, pEF-Myc-Akt3, pEF-Flag-Akt1-T308D, pEF-Flag-Akt1-S473D, pEF-Myc-Akt1-T308A-S473A, pEF-Myc-Akt1-T308D-S473D, pDs-Red-C1-Akt1, pDs-Red-C1-Akt2, pDs-Red-C1-Akt3, pDs-Red-Akt1-T308A-S473A, pDs-Red-Akt1-T308D-S473D, pEF-Flag-PTEN-Y138L, pEF-Myc-PTEN-Y138L, PHAGE-PTEN-G129E, pEF-Flag-PTEN-K221A-K223A-S226A, pEF-Myc-PTEN-K221A-K223A-S226A, PHAGE-PTEN-K221A-K223A-S226A, pEF-Flag-PDK1, pGEX4T-1-Akt1, pET-28a-PTEN, pGEX4T-1-PTEN, pGEX4T-1-PTEN-G129E, pGEX4T-1-PTEN-Y138L, pGEX4T-1-PTEN-C124S, pGEX4T-1-PTEN, pGEX4T-1-PTEN-S226A, pCMV-GST-PTEN, pCMV-GST-PTEN-S226A, pEF-Flag-PTEN-S226A, pEF-Flag-PTEN-S226D, pEF-Flag-PTEN (aa 1-185, aa 186-350, aa 1-350, aa 186-403), pEGFP-C1-PTEN (aa 351-403), pEF-Flag-Akt1 (aa 1-108, aa 108-480, aa 1-408, aa 108-408, aa 409-480), pEF-Flag-PP2A, pEF-Flag-PHLPP2, CFP-Akt1, CFP-Akt2, CFP-Akt3, CFP-PTEN, CFP-PTEN-K221A-K223A-S226A, YFP-Akt1, YFP-Akt2, YFP-Akt3, YFP-PTEN, and YFP-PTEN-K221A-K223A-S226A were constructed according to the manufacturer’s standard procedures.

**Reagents and antibodies**

The chemical reagents used in this study were listed as follows: wortmannin (9951, Cell Signaling), LY294002 (L9908, Sigma), recombinant human EGF (100-15, PeproTech), recombinant murine EGF (315-09, PeproTech), insulin (1342106, Sigma), Protein A/G (sc-2003, Santa Cruz), and recombinant inactive His-Akt1 (14-279, Upstate/Millipore). Anti-Akt1 antibody (4691), anti-Akt1 antibody (2920), anti-phospho-S473-Akt antibody (4060), anti-phospho-T308-Akt antibody (13038), anti-S6K antibody (9202), anti-phospho-T389-S6K antibody (9234), anti-GSK3β antibody (12456), anti-phospho-S9-GSK3β antibody (5558), anti-LMNB1 antibody (13435), anti-β-tubulin antibody (2128), anti-GAPDH antibody (5174), anti-ERp72 antibody (5033), anti-COXIV antibody (4850), anti-p110α antibody (4255), anti-p110βantibody (3011), anti-p85α antibody (13666), anti-PTEN antibody (9188), and anti-PTEN (sepharose bead conjugate) antibody (4326), secondary anti-rabbit antibody (5127), secondary anti-mouse antibody (91196), and anti-phospho-Akt substrate (RXXS*/T*) antibody (9614) were purchased from Cell Signaling Technology. Mouse anti-HA antibody ([H3663](http://www.sigmaaldrich.com/catalog/product/sigma/h3663)), rabbit anti-HA antibody ([H6908](http://www.sigmaaldrich.com/catalog/product/sigma/h6908)), mouse anti-Myc antibody (SAB2702192), rabbit anti-Myc antibody (C3956), mouse anti-Flag antibody ([F3165](http://www.sigmaaldrich.com/catalog/product/sigma/f4049)), rabbit anti-Flag antibody (F7425), and WGA-FITC (L4895) were purchased from Sigma. Anti-Flag M2 Affinity Gel (A2220) and 3× Flag Peptide (F4799) were purchased from Millipore. Anti-GST antibody (ab9085), anti-His antibody (ab5000), and anti-thiophosphate ester antibody (ab92570) were purchased from Abcam. Anti-PI(3, 4, 5)P3 beads (P-B345a) were purchased from Echelon Biosciences. Goat anti-mouse Alexa Fluor 555 (A-21422), goat anti-rabbit Alexa Fluor 555 (A-21428), goat anti-rabbit FITC (F-2765), goat anti-mouse FITC (F-2761), and goat anti-rabbit Alexa Fluor Plus 647 (A32733) were purchased from Invitrogen.

**Cell proliferation**

Cells (3×10^3^) were seeded on 96-well plates and treated with different stimulations. Then 10 µl substrate of the cell counting kit-8 (CCK-8, Dojindo Laboratories) was added into each well of the plate. The plates were incubated without light at 37 °C for 2 h. The optical density (OD) values were determined at an absorbance of 450 nm using an ELx800 microplate reader (BioTek).

**CRISPR-Cas9-mediated gene knockout assay**

The knockout of PI3K genes mediated by CRISPR-Cas9 is achieved by cloning the annealed single guide RNAs (sgRNAs) into lentiCRISPR V2 vector. The sgRNAs were designed according to the previous articles^2^ as follows: sghPIK3CA

Forward: 5’-CACCGCGAGCAGCACGAGGAAGATC-3’; Reverse: 5’-AAACGATCTTCCTCGTGCTGCTCGC-3’. sghPIK3CB Forward: 5’-CACCGGTGACAAGAAGTTGTGACCC-3’; Reverse: 5’-AAACGGGTCACAACTTCTTGTCACC-3’. sghPIK3R1 Forward: 5’-CACCGCTAGCATACCTCATCAGTAT-3’; Reverse: 5’-AAACATACTGATGAGGTATGCTAGC-3’.

**Immunoprecipitation (IP) and immunoblotting (IB) analysis**

For endogenous immunoprecipitation experiments, 293T cells (1×10^7^) were seeded on 10-cm dishes. After 24 h, the cells were lysed in lysis buffer (50 mM Tris-HCl pH 7.6, 150 mM NaCl, 0.5% Triton X-100, 2 mM EGTA, 10 mM NaF, 1 mM Na_3_VO_4_, 2 mM DTT and 1× Cocktail). The whole cell extracts were re-cleared with the centrifugation at 12000 rpm for 10 min and were incubated overnight with anti-Akt or anti-PTEN antibody and 30 μl Protein A/G beads (Santa Cruz) at 4 °C. Beads were washed three times using lysis buffer and boiled for 5 min after adding 2x Laemmli buffer. Then samples were subjected to Western Blotting assay and analyzed with corresponding antibodies according to the instructions as indicated^1^. For exogenous immunoprecipitation experiments, 293T cells (5×10^6^) were seeded on 10-cm dishes and transfected with a total of 12 μg plasmids. Then cell lysates were incubated overnight with 30 μl anti-Flag agarose (Millipore) at 4 °C. After washing three times with lysis buffer, beads were boiled with 2× Laemmli buffer for 5 min and subjected to IB analysis. We used rabbit antibody for IP and used mouse secondary antibody for detection (or vice versa), or used special secondary antibody that could not recognize the heavy chain of denatured IgG (anti-rabbit, 5127, CST; anti-mouse, 91196, CST).

**Immunofluorescence (IF) assay**

293T cells (3×10^4^) were seeded on confocal dishes for 48 h, fixed with 4% formaldehyde for 15 min, and incubated with 0.3% Triton X-100 for 10 min at room temperature. After washing three times, cells were blocked with 5% BSA for 30 min. The samples were incubated with primary antibodies overnight at 4 °C and then washed three times using PBS containing 0.1% Tween-20. Next, cells were incubated with appropriate second antibodies for 1 h at 37 °C, and the nucleus was stained with 4, 6-diamidino-2-phenylindole (DAPI) for 5 min. Fluorescence images were captured using Leica (SP8-STED) confocal microscope.

**Subcellular fractionation assay**

Nuclear-cytosolic fractionation extracts were performed using a nuclear-cytoplasmic extraction kit (Applygen Technologies) according to the manufacturer’s instructions. For nuclear-membrane fractionation assays, 293T cells were collected and suspended in hypotonic buffer (10 mM Tris-HCl pH 7.4, 10 mM KCl and 1.5 mM MgCl_2_), incubated on ice for 30 min, and centrifuged at 2,500 rpm for 5 min at 4 °C. Then the precipitations were washed with hypotonic buffer supplemented with 0.3% NP-40 for three times, the nuclear extracts were resuspended in lysis buffer and boiled with 5× Laemmli buffer for 5 min and subjected to IB analysis. And the supernatants were centrifuged at 100,000 xg for 1 h at 4 °C, the membrane precipitations were resuspended in lysis buffer and boiled with 5× Laemmli buffer for 5 min and subjected to IB analysis.

**Protein purification and GST pull-down assay**

The human Akt1 gene was cloned into pGEX-4T-1 expression plasmid containing GST-tag, and the human PTEN gene was cloned into pET-28a expression plasmid containing His-tag. Recombinant GST-Akt1 and His-PTEN proteins were expressed in BL21 E. coli and purified as described previously.^1^ The quality of proteins was examined with IB analysis and Coomassie brilliant blue staining. His-PTEN protein was incubated with anti-GST beads that were equilibrated using NETN buffer (100 mM NaCl, 1 mM EDTA, 20 mM Tris-HCl pH 7.4, 0.5% NP-40). Then pre-cleared His-PTEN was mixed with GST or GST-Akt1 protein respectively, and incubated overnight with an equal amount of anti-GST beads at 4 °C. The samples were washed five times with NETN buffer and then analyzed by IB analysis.

***In vitro* protein phosphatase assay**

24 h post-transfection with indicated plasmids, the cells were lysed in the lysis buffer (50 mM Tris-HCl pH 7.6, 150 mM NaCl, 0.5% Triton X-100, 2 mM EGTA, 10 mM NaF, 1 mM Na_3_VO_4_, 2 mM DTT and 1× Cocktail). The cell extracts were pre-cleared with centrifugation at 12000 g for 10 min and then immunoprecipitated with anti-Flag agarose or indicated antibody plus protein A/G. The immunoprecipitates were eluted with 3× Flag peptide at 200 µg/ml. GST-PTEN, GST-PTEN-G129E, GST-PTEN-Y138L, and GST-PTEN-C124S proteins were purified from BL21 E. coli. For the phosphatase assays, immunoprecipitates or purified proteins were rotationally incubated at 37 °C for 2 h in phosphatase activity assay buffer (50 mM Tris-HCl pH 7.6, 10 mM MgCl_2_ and 2 mM DTT), and then the phosphorylation of Akt1 was detected by IB analysis.

***In vitro* protein cold kinase assay**

As described in i*n vitro* protein phosphatase assays, the protein samples were prepared. For the kinase assays, immunoprecipitates were rotationally incubated for 20 min at 37 °C in 100 μl kinase reaction buffer (25 mM Tris-HCl pH 7.5, 10 mM MgCl_2_, 5 mM β-Glycerophosphate, 2 mM DTT, and 0.1 mM Na_3_VO_4_) containing 200 μM ATP and 100 ng recombinant 6×His-Akt1 protein *in vitro*, and then the phosphorylation of Akt1 was detected by IB analysis.

***In vitro* modified protein kinase assay**

293T cells were transfected with Flag-Myr-Akt1 or vector, and after stimulating with insulin, the cells were lysed and immunoprecipitated with anti-Flag agarose; then Flag-Myr-Akt1 was eluted with 3× Flag peptide at 200 µg/ml. 1µg of the bacterially purified GST-PTEN or GST-PTEN-S226A fusion proteins were incubated with Flag-Myr-Akt1 or vector control sample in the presence of 200 μM ATP (with 100 ng [γS] ATP (Abcam, 138911)) in the kinase reaction buffer (50mM Tris pH7.5, 10 mM MgCl_2_, 2 mM DTT) for 30 minutes at 30°C. The tube was shaken once/10min. The reaction was subsequently stopped by adding EDTA at final concentration of 0.1 mM, and further incubated for another 1 hour by adding the pNitrobenzyl mesylate (Abcam, 138910) to alkylate the thiophosphorylation site on the substrates. The reaction was stopped by adding 2× SDS loading buffer and resolved by SDS-PAGE.

**FRET assay**

293T cells (3×10^4^) were seeded on confocal dishes and transfected with CFP or YFP fusional plasmids for 48 h. Live cell imaging was performed by confocal Zeiss LSM880 microscope. CFP was excited by 405 nm laser source and emission was detected in the range of 460-492 nm; YFP was excited by 514 nm laser source and emission was detected in the range of 526-589 nm; YFP was photobleached by a 514 nm laser source at 100% power for 1 min. Data were collected from cells in 6 different regions of the same confocal dish. CFP fluorescence and YFP fluorescence of representative regions before or after photobleaching were obtained using respective filter sets. Quantitative analysis was carried out by ZEN software (Zeiss).

**ELISA**

PIP3 levels were detected by ELISA kit (Echelon) according to the manufacturer’s instructions.

**Animal experiments**

All animal experiments were conducted in accordance with the Regulations of Guangdong Province Laboratory Animal Management and approved by the Sun Yat-sen University Animal Experiment Ethics Committee. This study is compliant with all relevant ethical regulations regarding animal research.

Mouse xenograft assays. Two PTEN deficiency cell lines, prostate cancer cells PTEN-depleted PC3 and glioma-derived cells U87MG (3×10^5^) stably expressing vector, PTEN, PTEN-G129E, or PTEN-3m, were respectively mixed with 50 μl sterile PBS and subcutaneously injected into 4-week-old female [BALB/c nude](https://www.sciencedirect.com/topics/immunology-and-microbiology/balb-c-nude-mouse) mice (*n* = 3 per group). After 6 days, the tumor size was measured every two days using vernier caliper. The tumor volume was determined by formula *L* x *W*^2^ x 0.5, in which *L* is the longest diameter and *W* is the shortest diameter. 16 days later, mice were euthanized to dissect solid tumors, then tumor weights were measured and recorded. After homogenizing the tumor tissues, the expression of proteins was analyzed by IB analysis with the indicated antibodies.

**The orthotopic implantation assays**. PTEN-depleted U87MG cells (1×10^5^) stably expressing PTEN, PTEN-G129E or PTEN-3m were injected into 4-week-old female [BALB/c nude](https://www.sciencedirect.com/topics/immunology-and-microbiology/balb-c-nude-mouse) mice (*n* = 8 per group) at 2.5 mm below the skull, 2 mm lateral and 0.5 mm anterior to the bregma. 14 days later, two mice from every group were randomly selected to be killed for H&E staining of a brain cross-section. The survival time of other mice (*n* = 6 per group) was monitored until all of the mice were dead, and then a Kaplan-Meier survival curve of mice was performed using the GraphPad [Prism](https://www.sciencedirect.com/topics/agricultural-and-biological-sciences/prisms) 6 software.

**Conditional Pten deficiency mice assays**. Generation of mice with tamoxifen-induced conditional Pten deficiency have been described previously.^1^ The mice were fed with sterilized water and food. 6-week-old female mice were used in this experiment. Conditional Pten knockout mice and WT mice were starved for 18 h, intravenously injected with PBS or wortmannin (0.7 mg/kg) for 2 h, and then injected intraperitoneally with insulin (0.5 U/kg).

**Statistical analysis**

Data are expressed as means ± SEM. The significant differences between samples were tested by Student’s *t*-test or two-way ANOVA analysis using GraphPad Prism 6 software. Survival analysis was analyzed by the Kaplan-Meier method. The values of *P* < 0.05 are considered to have statistical significance.

**References**

1. Li, S. *et al.* The tumor suppressor PTEN has a critical role in antiviral innate immunity. *Nat. Immunol.* **17**, 241-249 (2016).

2. Shalem, O. *et al.* Genome-scale CRISPR-Cas9 knockout screening in human cells. *Science* **343**, 84-87 (2014).

**Supplementary Figure Legends**

**Figure. S1 PTEN directly interacts with Akt. a** 293T cells were co-transfected with HA-PTEN and vector or Flag-Akt1, or co-transfected with Myc-Akt1 and vector or Flag-PTEN. The whole cell extracts were collected and subjected to IP and IB analysis. **b** Purified recombinant His-PTEN proteins were mixed with GST or GST-Akt1 proteins as indicated, pulled down with anti-GST beads, and subjected to IB analysis. **c** The immunofluorescence (IF) analysis was performed to detect endogenous PTEN or Akt in 293T cells. Scale bar is 10 μm. **d** 293T cells were transfected with RFP-Akt1 and GFP-PTEN. The cells were scanned at different levels by confocal microscopy and the 3D reconstruction of images were shown as indicated. Scale bar is 10 μm. **e** 293T cells were co-transfected with Myc-Akt1 and vector, Flag-PTEN, or Flag-PTEN-truncated mutants, or co-transfected with GFP-PTEN-C tail and vector or Flag-Akt1 as indicated. The whole cell extracts were collected and subjected to IP and IB analysis. **f** 293T cells were co-transfected with HA-PTEN and vector, Flag-Akt1, or Flag-Akt1-truncated mutants as indicated. The whole cell extracts were collected and subjected to IP and IB analysis. **g** 293T cells were co-transfected with Myc-Akt1 and vector or Flag-PTEN, the whole cell extracts were first IP with or without PIP3 beads, then the cell lysis were second IP with Flag beads, followed by IB analysis. **h** WT 293T cells or PI3K-KO 293T cells were co-transfected with Myc-Akt1 and vector or Flag-PTEN. The whole cell extracts were collected and subjected to IP and IB analysis. **i** 293T cells were transfected with Flag-PTEN. Then whole cell extracts were collected and subjected to IP and IB analysis. **j** The whole cell extracts of HCT116 cells were collected and subjected to IP and IB analysis. HCT116 cells were co-transfected with Myc-Akt1 and Flag-PTEN. The cells were subjected to IF assays. Scale bar, 10 μm. **k** 293T cells were co-transfected with HA-PTEN and vector, Flag-Akt1, Flag-Akt2, or Flag-Akt3. The whole cell extracts were subjected to IP followed by IB analysis. **l** 293T cells were co-transfected with Myc-Akt2 and vector or Flag-PTEN, or co-transfected with Myc-Akt3 and vector or Flag-PTEN. The whole cell extracts were subjected to IP followed by IB analysis. **m** 293T cells were transfected with RFP-Akt2 or RFP-Akt3 alone or together with GFP-PTEN. The cells were subjected to IF assays. Scale bar, 10 μm. All experiments were performed at least two times independently.

**Figure. S2 The interactions between PTEN and Akt were detected by FRET. a** Schematic diagrams of the detection of the interaction between protein A and protein B using acceptor photobleaching FRET assays. **b** The quantitative analysis method of CFP changes after YFP photobleaching. **c** 293T cells were transfected with CFP-YFP fusion plasmid or co-transfected with CFP and YFP. The cells were subjected to FRET assays (the red boxes were bleached, the green boxes were not bleached, and the blue boxes were negative control). Scale bar, 10 μm. **d** The quantifications of CFP changes after YFP photobleaching. Data were performed three times independently and represented as mean ± SEM. ***P* < 0.01, based on the Student’s *t*-tests. **e** 293T cells were transfected with vector, CFP-Akt1, CFP-Akt2, CFP-Akt3, YFP-Akt1, YFP-Akt2, or YFP-Akt3. The cells were subjected to IB analysis. **e** 293T cells were transfected with vector, CFP-PTEN, or YFP-PTEN. The cells were subjected to IB analysis. **f** 293T cells were co-transfected with CFP-PTEN and YFP-PTEN. The cells were subjected to FRET assays (the red boxes were bleached, the green boxes were not bleached, and the blue boxes were negative control). Scale bar, 10 μm. **g** The quantifications of CFP changes after YFP photobleaching. Data were performed three times independently and represented as mean ± SEM. ***P* < 0.01, based on the Student’s *t*-tests. **h** 293T cells were co-transfected with CFP-PTEN and YFP-Akt1, CFP-Akt1 and YFP-PTEN, CFP-PTEN and YFP-Akt2, CFP-Akt2 and YFP-PTEN, CFP-PTEN and YFP-Akt3, or CFP-Akt3 and YFP-PTEN. The cells were subjected to FRET assays (the red boxes were bleached, the green boxes were not bleached, and the blue boxes were negative control). Scale bar, 10 μm. **i** The quantifications of CFP changes after YFP photobleaching were shown. Data were performed three times independently and represented as mean ± SEM. ***P* < 0.01; ****P* < 0.001, based on the Student’s *t*-tests. All experiments were performed at least two times independently.

**Figure. S3 PTEN can be phosphorylated at S226 site by Akt1. a** 293T cells were co-transfected with GST-PTEN or GST-PTEN-S226A and vector, or Flag-Myr-Akt1. The whole cell extracts were collected and subjected to IP and IB analysis. **b** WT 293T cells or Akt1^-/-^ 293T cells were transfected with GST-PTEN, then the whole cell extracts were collected and subjected to IP and IB analysis. **c** 293T cells were transfected with vector, or Flag-Myr-Akt1 respectively, stimulated with insulin and immunoprecipitated with anti-Flag agarose, then eluted with 3× Flag peptide. GST tag proteins were purified from bacterial. The purified proteins were subjected to kinase assay. **d** 293T cells were co-transfected with Myc-Akt1 and vector, Flag-PTEN, Flag-PTEN-S226A, or Flag-PTEN-S226D. The whole cell extracts were collected and subjected to IP and IB analysis.

**Figure. S4 The interaction between PTEN and Akt was enhanced when cells were stimulated by insulin, EGF or SeV. a** 293T cells were serum-starved for 18 h before treated with insulin (1 μg/ml) or EGF (100 ng/ml) and subjected to IP and IB analysis. **b** 293T cells were stimulated with SeV and subjected to IP and IB analysis. **c** 293T cells were co-transfected with HA-PTEN and vector or Flag-Akt1. Cells were serum-starved for 18 h before treated with insulin (1 μg/ml) or EGF (100 ng/ml) and subjected to IP and IB analysis. **d** 293T cells were co-transfected with HA-PTEN and vector or Flag-Akt1. Cells were stimulated with SeV and subjected to IP and IB analysis. **e** 293T cells were co-transfected with CFP-PTEN and YFP-Akt1, or CFP-Akt1 and YFP-PTEN. Cells were serum-starved for 18 h, then above untreated or treated with insulin (1 μg/ml) or EGF (100 ng/ml) for 10 min before being subjected to FRET assays (the red boxes were bleached, the green boxes were not bleached, and the blue boxes were negative control). Scale bar is 10 μm. **f** 293T cells were co-transfected with CFP-PTEN and YFP-Akt1, or CFP-Akt1 and YFP-PTEN. Cells were above untreated or treated with SeV for 10 h, then subjected to FRET assays (the red boxes were bleached, the green boxes were not bleached, and the blue boxes were negative control). Scale bar is 10 μm. **g** The quantification of CFP in (**e**) and (**f**) after YFP photobleaching. Data were performed three times independently and represented as mean ± SEM. **P* < 0.05; ***P* < 0.01; ****P* < 0.001, based on the Student’s *t*-tests. All experiments were performed at least two times independently.

**Figure. S5 PTEN inhibits the recruitment of Akt to the plasma membrane induced by insulin or EGF. a** WT, *Pten^-/-^*, and *Pten^+/+^* MEFs were serum-starved for 18 h, then treated with insulin (1 μg/ml) or EGF (100 ng/ml) for 10 min, and subjected to IF assays. Scale bar is 10 μm. **b** 293T cells were co-transfected with Myc-Akt1 and vector, Flag-PTEN, Flag-PTEN-G129E, Flag-PTEN-Y138L, or Flag-PTEN-C124S. The cells were serum-starved for 18 h, and then treated with insulin (1 μg/ml) or EGF (100 ng/ml) for 10 min. Finally, the nucleus and cytomembrane fractions were extracted and subjected to IB analysis. **c, d** PTEN-depleted PC3 cells were transfected with Flag-PTEN, Flag-PTEN-G129E, Flag-PTEN-Y138L, or Flag-PTEN-C124S respectively, or co-transfected with Flag-PTEN-G129E and Myc-PTEN-Y138L. The cells were serum-starved for 18 h, and then treated with insulin (1 μg/ml) (**c**) or EGF (100 ng/ml) (**d**) for 10 min before being subjected to IF assays. Scale bar, 10 μm. **e, f** 293T cells were transfected with RFP-Akt2 or RFP-Akt3 alone, or together with GFP-PTEN. The cells were serum-starved for 18 h, and then subjected to IF assays 10 min after the treatment of insulin (1 μg/ml) (**e**) or EGF (100 ng/ml) (**f**). Scale bar, 10 μm. All experiments were performed at least two times independently.

**Figure. S6 PTEN reduces the phosphorylation of Akt1 in the PI3K inhibited cells.**

**a** PTEN-depleted PC3 cells were transfected with vector, Myc-PTEN, or Myc-PTEN-C124S or co-transfected with the aforementioned plasmids and Flag-Akt1. The cells were treated with wortmannin (1 µM) for 2 h and subjected to IB analysis. **b** 293T cells were transfected with scramble shRNA or shRNA targeting PTEN (shPTEN), or co-transfected with the aforementioned shRNAs and Flag-Akt1.The cells were treated with wortmannin (1 µM) for 2 h and then subjected to IB analysis. **c, d** *Pten^+/+^* and *Pten^-/-^* MEFs were serum-starved for 18 h, treated with wortmannin (1 µM) (**c**) or LY294002 (5 µM) (**d**) for 2 h, and then stimulated with insulin (1 μg/ml) or EGF (100 ng/ml) as indicated. The whole cell extracts were collected and subjected to IB analysis. **e** PTEN-wild-type mice and PTEN-mutant mice were starved for 18 h, injected intravenously with PBS or wortmannin (0.7 mg/kg) for 2 h, and then injected intraperitoneally with insulin (0.5 U/kg) as indicated. Liver tissues were obtained and subjected to IB analysis. All experiments were performed at least two times independently.

**Figure. S7 PTEN reduces the phosphorylation of Akt1 in the PI3K deficient cells. a** The monoclonal PI3K knockout (PIK3CA^-/-^ PIK3CB^-/-^ PIK3R1^-/-^) 293T cells were detected by T7E1 enzyme assays. **b** The sequencing results of the gene sequences of PIK3CA, PIK3CB, or PIK3R1 in monoclonal PI3K knockout 293T cells. **c** WT or PI3K knockout 293T cells were transfected with vector, Flag-PTEN, or Flag-PTEN-C124S. The cells were subjected to IB analysis. **d** PI3K knockout 293T cells were transfected with Flag-TBK1 or Flag-IKKε alone or together with Myc-PTEN or Myc-PTEN-C124S. The whole cell extracts were subjected to IB analysis. **e** PI3K knockout 293T cells were co-transfected with scramble shRNA or shPTEN and Flag-TBK1 or Flag-IKKε. 48 h post-transfection, the whole cell extracts were subjected to IB analysis. All experiments were performed at least two times independently.

**Figure. S8 PTEN can directly dephosphorylate Akt1 at S473 and T308 by its protein phosphatase activity. a** 293T cells were transfected with Myc-Akt1 and vector, Flag-PTEN, Flag-PTEN-G129E, Flag-PTEN-Y138L, or Flag-PTEN-C124S. The whole cell extracts were subjected to IP and IB analysis. **b, c** 293T cells were transfected with vector, Flag-PTEN, Flag-Akt1, Flag-PTEN-Y138L (**b**), or Flag-PTEN-C124S (**c**) respectively, immunoprecipitated with anti-Flag agarose, and eluted with 3× Flag peptide. The purified proteins were subjected to phosphatase assays. **d** Flag-Myr-Akt1 proteins were purified from 293T cells, and eluted with 3× Flag peptide. GST, GST-PTEN, GST-PTEN-G129E, GST-PTEN-Y138L, GST-PTEN-C124S proteins were purified from E. coil. The purified proteins were subjected to phosphatase assays. **e** 293T cells were transfected with Myc-Akt1 and vector, Flag-PTEN, or Flag-PP2A. The whole cell extracts were subjected to IP and IB analysis. **f** 293T cells were transfected with Myc-Akt1 and vector, Flag-PTEN, or Flag-PHLPP2. The whole cell extracts were subjected to IP and IB analysis. **g**, **h** 293T cells were transfected with RFP-Akt1-T308A-S473A (**g**) or RFP-Akt1-T308D-S473D (**h**) alone, or together with Flag-PTEN, Flag-PTEN-G129E, Flag-PTEN-Y138L, or Flag-PTEN-C124S. The cells were serum-starved for 18 h, and then treated with insulin (1 μg/ml) for 10 min before being subjected to IF assays. Scale bar is 10 μm. All experiments were performed at least two times independently.

**Figure. S9 PTEN-G129E inhibits the phosphorylation of Akt1 at S473 and T308 activated by classical or non-classical pathway. a** 293T cells were transfected with Flag-PDK1, Flag-mTOR, or Flag-Rictor alone or together with Myc-PTEN-G129E or Myc-PTEN-C124S. The whole cell extracts were subjected to IB analysis. **b** 293T cells were transfected with Flag-TBK1 or Flag-IKKε alone or together with Myc-PTEN-G129E or Myc-PTEN-C124S. The whole cell extracts were subjected to IB analysis. **c, d** PC3 cells stably expressing vector or PTEN-G129E were serum-starved for 18 h, and then subjected to IB after being stimulated with insulin (1 μg/ml) (**c**) or EGF (100 ng/ml) (**d**) for the indicated time. **e-i** 293T cells were transfected with vector, Flag-PTEN, Flag-PTEN-G129E, Flag-PDK1 (**e**), Flag-mTOR (**f**), Flag-Rictor (**g**), Flag-TBK1 (**h**), or Flag-IKKε (**i**). The whole cell extracts were subjected to IP with anti-Flag agarose. The Flag-tagged proteins were eluted with 3× Flag peptide and subjected to kinase assays. All experiments were performed at least two times independently.

**Figure. S10 PTEN with different subcellular localizations interact with Akt1 and decrease the phosphorylation of Akt1 at S473 and T308. a** Structural schematic diagram of PTEN fused with different subcellular localization sequences. **b** The subcellular localizations of various PTENs described in (**a**) were examined using IF assays. Scale bar, 10 μm. **c** PC3 cells were transfected with vector, Flag-PTEN, Flag-NLS-PTEN, Flag-ER-PTEN, Flag-Mito-PTEN, or Flag-Myr-PTEN. The whole cell extracts were subjected to IB analysis. **d** 293T cells were co-transfected with Myc-Akt1 and vector, Flag-PTEN, Flag-NLS-PTEN, Flag-ER-PTEN, Flag-Mito-PTEN, or Flag-Myr-PTEN. The whole cell extracts were subjected to IP and IB analysis with indicated antibodies. **e** 293T cells were transfected with Flag-ER-PTEN or Flag-Mito-PTEN alone or together with RFP-Akt1. The cells were subjected to IF assays. Scale bar, 10 μm. **f, g** PC3 cells were transfected with vector, Flag-ER-PTEN, or Flag-Mito-PTEN. The cells were serum-starved for 18 h, and then treated with insulin (1 μg/ml) (**f**) or EGF (100 ng/ml) (**g**) for indicated time before being subjected to IB analysis. **h** 293T cells were co-transfected with RFP-Akt1 and Flag-ER-PTEN or Flag-Mito-PTEN. The cells were serum-starved for 18 h, and then treated with insulin (1 μg/ml) or EGF (100 ng/ml) for 10 min before being subjected to IF assays. Scale bar, 10 μm. All experiments were performed at least two times independently.

**Figure. S11 Nuclear PTEN directly dephosphorylates nuclear Akt1 at S473 and T308. a** PC3 cells were transfected with vector, Flag-PTEN, or Flag-PTEN-C124S. The nucleus/cytoplasm fractions were extracted and then subjected to IB analysis. **b** PC3 cells were transfected with vector or Flag-NLS-PTEN. The nucleus/cytoplasm fractions were extracted and then subjected to IB analysis. **c** 293T cells were co-transfected with Myc-Akt1 and vector or Flag-NLS-PTEN, or co-transfected with Flag-NLS-PTEN and vector or Myc-Akt1. Followed by IP and IB analysis. **d** 293T cells were transfected with Flag-NLS-PTEN alone or together with RFP-Akt. The cells were subjected to IF assays. Scale bar, 10 μm. **e** 293T cells were transfected with Flag-Akt1 and HA-PTEN separately or together. Nuclear and cytoplasmic fractions were extracted and subjected to IP and IB analysis. **f** PC3 cells were transfected with vector or Flag-NLS-PTEN. The cells were serum-starved for 18 h, and then treated with insulin (1 μg/ml) or EGF (100 ng/ml) as indicated before being subjected to IB analysis. All experiments were performed at least two times independently.

**Figure. S12 Mutation in the Akt-interacting motif of PTEN attenuates the inhibitory effect of PTEN on the phosphorylation of Akt1. a** In the absence or presence of Myc-Akt1, PC3 cells were transfected with vector, Flag-PTEN, or Flag-PTEN-3m. The cells were left untreated or treated with wortmannin (1 µM) for 2 h, and then subjected to IB analysis. **b** WT or PI3K knockout 293T cells were transfected with vector, Flag-PTEN, or Flag-PTEN-3m. The cells were subjected to IB analysis. **c** 293T cells were co-transfected with CFP-PTEN and YFP-Akt1, CFP-Akt1 and YFP-PTEN, CFP-PTEN-3m and YFP-Akt1, or CFP-Akt1 and YFP-PTEN-3m. The cells were subjected to FRET assays (the red boxes were bleached, the green boxes were not bleached, and the blue boxes were negative control). Scale bar is 10 μm. **d** The quantifications of CFP after YFP photobleaching were shown. Data were performed three times independently and represented as mean ± SEM. **P* < 0.05; ****P* < 0.001, based on the Student’s *t*-tests. All experiments were performed at least two times independently.

**Figure. S13 PTEN inhibits tumorigenesis by directly dephosphorylating Akt1. a** PTEN-depleted U87MG cells or PC3 cells stably expressing vector, PTEN, PTEN-G129E, or PTEN-3m were left untreated or stimulated with or EGF. The PIP3 in the cells were extracted and examined with ELISA. Ns indicates nonsignificant (*P* ＞0.05). **b** 293T cells were transfected with Flag-PTEN or Flag-PTEN-3m, then the whole cell extracts were subjected to IP with PIP3 beads and IB analysis. **c** PTEN-depleted U87MG cells or PC3 cells stably expressing vector, PTEN, PTEN-G129E, or PTEN-3m were seeded in 96-well plates, and cell viability was determined by CCK-8 at different times as indicated. Data were performed three times independently and represented as mean ± SEM. ****P* < 0.001, based on the two-way ANOVA. **c** Lysates of dissected tumor tissues were subjected to IB analysis. **d** DLD1-Akt1/2^-/-^ cells were stably expressed with vector, PTEN, or PTEN-3m, then cells were seeded in 96-well plates, and cell viability was determined by CCK-8 at different times as indicated. Data were performed three times independently and represented as mean ± SEM. Ns indicates nonsignificant (*P* ＞0.05). **e** Lysates of dissected tumor tissues were subjected to IB analysis. **f, g** PTEN-depleted U87MG cells stably expressing vector, PTEN, PTEN-G129E, or PTEN-3m were injected into the brain of mice (*n* = 8 per group) in situ. 14 days later, two mice from every group were randomly selected to be euthanized. The brain tissues were collected for H&E staining (**f**). The survival times of other mice (*n* = 6 per group) were monitored until the endpoint of the experiment, and then survival analysis of these mice was performed (**g**). Data were represented as mean ± SEM. ****P* < 0.001, based on the Log-rank (Mantel-Cox) test. The experiments in b were performed two times independently with similar results. The experiments in a and c-d were performed three times independently.
