# Supplementary material for: PTEN suppresses tumorigenesis by directly dephosphorylating Akt
Source: Signal Transduct Target Ther. 2021 Jul 12;6:262. doi: 10.1038/s41392-021-00571-x (PMC8273154; doi:10.1038/s41392-021-00571-x)
Supplement: Supplementary file 2 — Supplementary Figures [file 41392_2021_571_MOESM2_ESM.pdf]

Figure S1

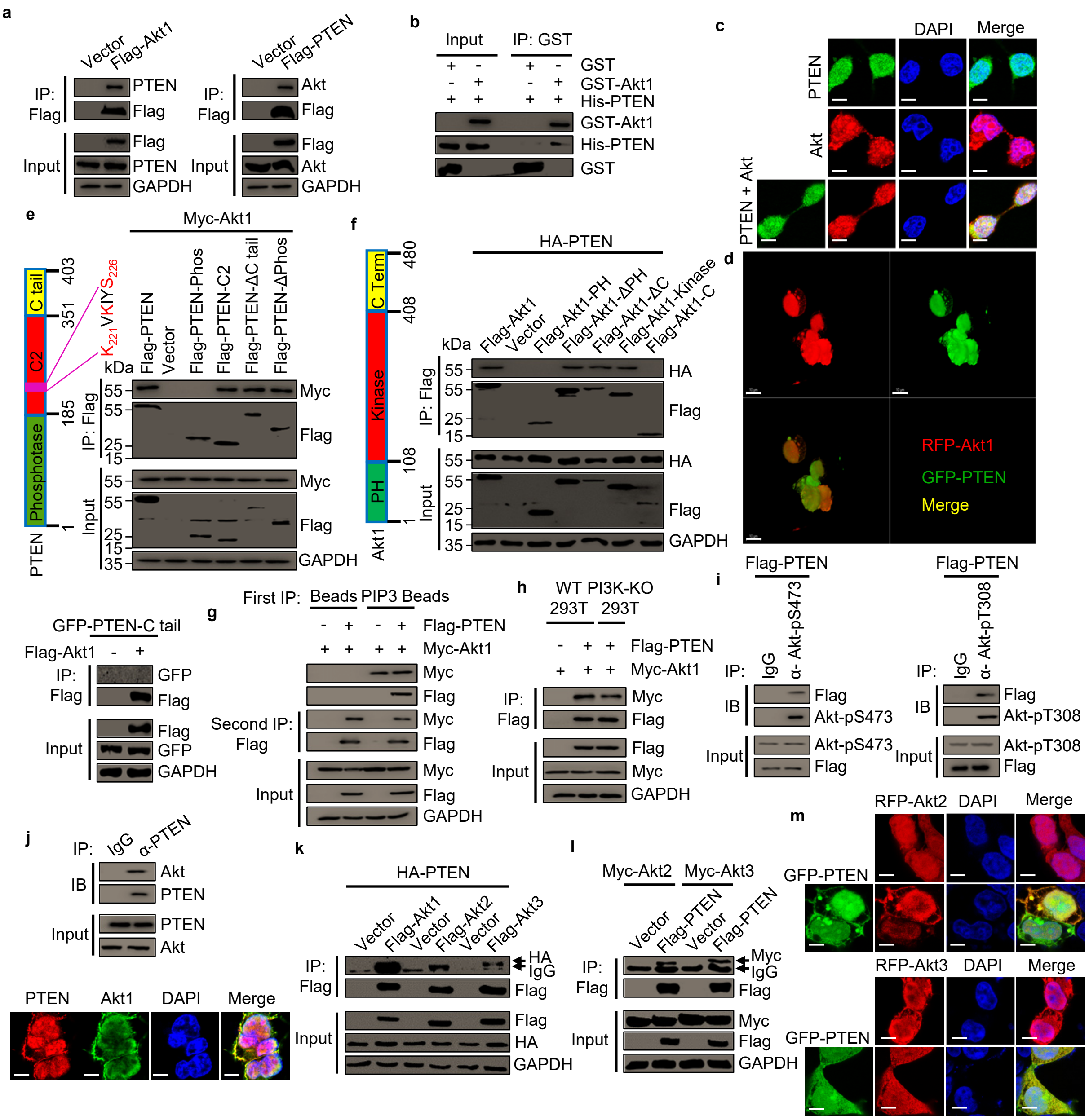

a
Vector
Flag-Akt1
IP: Flag
PTEN
Flag
Input
Flag
PTEN
GAPDH
Vector
Flag-PTEN
Akt
Flag
Akt
GAPDH
b
Input
IP: GST
GST
GST-Akt1
His-PTEN
GST-Akt1
His-PTEN
GST
c
DAPI
Merge
PTEN
Akt
PTEN + Akt
d
RFP-Akt1
GFP-PTEN
Merge
e
Myc-Akt1
C tail
C2
Phosphotase
PTEN
403
351
185
1
K221VKIYS226
kDa
Flag-PTEN
Vector
Flag-PTEN-Phos
Flag-PTEN-C2
Flag-PTEN-ΔC tail
Flag-PTEN-ΔPhos
IP: Flag
Input
Myc
Flag
GAPDH
f
HA-PTEN
C Term
Kinase
PH
Akt1
480
408
108
1
Flag-Akt1
Vector
Flag-Akt1-PH
Flag-Akt1-ΔPH
Flag-Akt1-ΔC
Flag-Akt1-Kinase
Flag-Akt1-C
HA
Flag
GAPDH
GFP-PTEN-C tail
Flag-Akt1
GFP
g
First IP:
Beads
PIP3 Beads
Flag-PTEN
Myc-Akt1
Second IP: Flag
Myc
h
WT 293T
PI3K-KO 293T
i
Flag-PTEN
IgG
α- Akt-pS473
α- Akt-pT308
IB
Akt-pS473
Akt-pT308
j
IgG
α-PTEN
Akt1
k
Flag-Akt2
Flag-Akt3
IgG
l
Myc-Akt2
Myc-Akt3
Vector
Flag-PTEN
m
RFP-Akt2
RFP-Akt3
GFP-PTEN

Figure S2

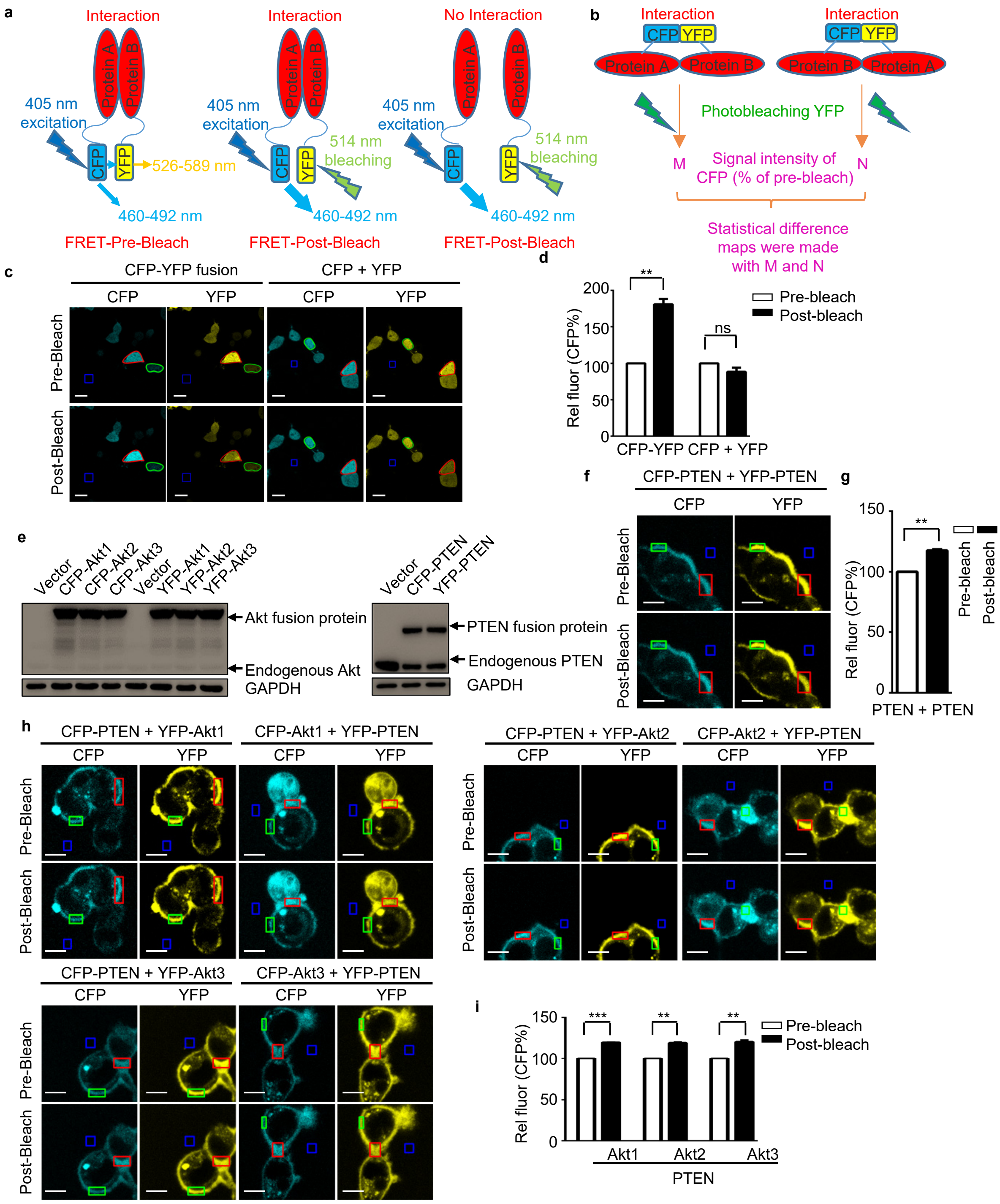

a
Interaction
Protein A
Protein B
405 nm excitation
CFP
YFP
526-589 nm
460-492 nm
FRET-Pre-Bleach
Interaction
514 nm bleaching
FRET-Post-Bleach
No Interaction
b
Interaction
Protein A
Protein B
Photobleaching YFP
M
N
Signal intensity of CFP (% of pre-bleach)
Statistical difference maps were made with M and N
c
CFP-YFP fusion
CFP + YFP
Pre-Bleach
Post-Bleach
d
Rel fluor (CFP%)
Pre-bleach
Post-bleach
**
ns
CFP-YFP CFP + YFP
e
Vector
CFP-Akt1
CFP-Akt2
CFP-Akt3
YFP-Akt1
YFP-Akt2
YFP-Akt3
Akt fusion protein
Endogenous Akt
GAPDH
CFP-PTEN
YFP-PTEN
PTEN fusion protein
Endogenous PTEN
f
CFP-PTEN + YFP-PTEN
g
PTEN + PTEN
h
CFP-PTEN + YFP-Akt1
CFP-Akt1 + YFP-PTEN
CFP-PTEN + YFP-Akt2
CFP-Akt2 + YFP-PTEN
CFP-PTEN + YFP-Akt3
CFP-Akt3 + YFP-PTEN
i
***
Akt1
Akt2
Akt3
PTEN

Figure S3

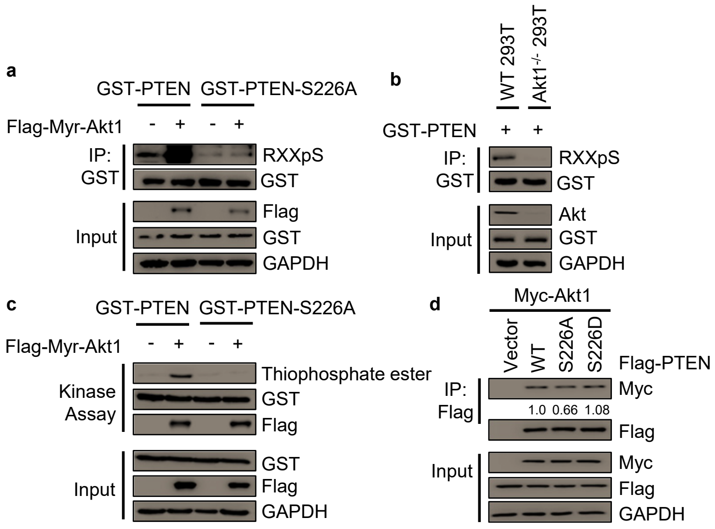
a
GST-PTEN
GST-PTEN-S226A
Flag-Myr-Akt1
IP: GST
RXXpS
GST
Input
Flag
GST
GAPDH
b
WT 293T
Akt1-/- 293T
GST-PTEN
IP: GST
RXXpS
GST
Input
Akt
GST
GAPDH
c
GST-PTEN
GST-PTEN-S226A
Flag-Myr-Akt1
Kinase Assay
Thiophosphate ester
GST
Flag
Input
GST
Flag
GAPDH
d
Myc-Akt1
Vector
WT
S226A
S226D
Flag-PTEN
IP: Flag
Myc
1.0
0.66
1.08
Flag
Input
Myc
Flag
GAPDH

Figure S4

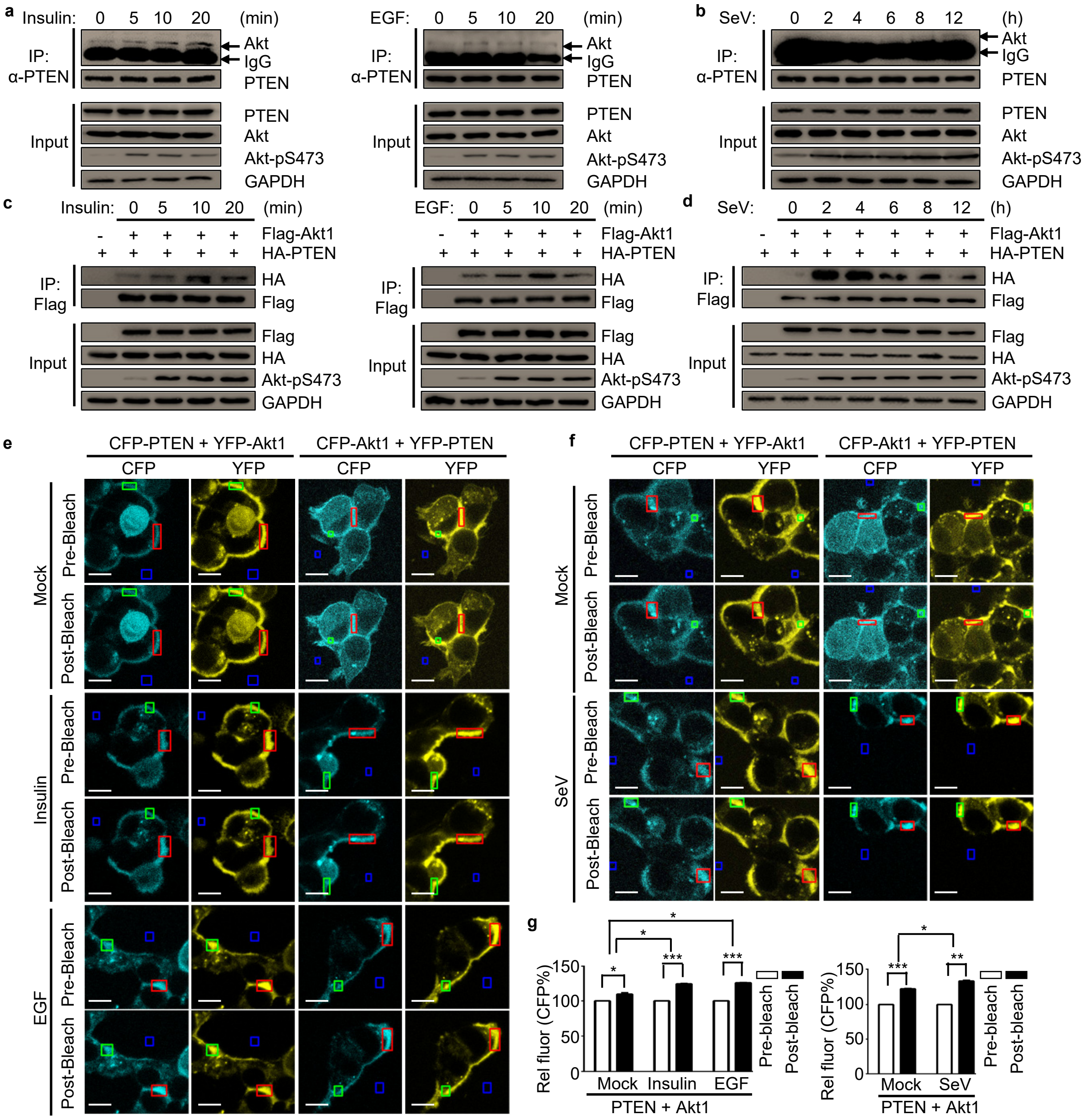

a
Insulin: 0 5 10 20 (min)
EGF: 0 5 10 20 (min)
IP: α-PTEN
Akt
IgG
PTEN
Input
PTEN
Akt
Akt-pS473
GAPDH
b
SeV: 0 2 4 6 8 12 (h)
c
Insulin: 0 5 10 20 (min)
EGF: 0 5 10 20 (min)
Flag-Akt1
HA-PTEN
IP: Flag
HA
Flag
d
SeV: 0 2 4 6 8 12 (h)
e
CFP-PTEN + YFP-Akt1
CFP-Akt1 + YFP-PTEN
CFP
YFP
Pre-Bleach
Post-Bleach
Mock
Insulin
EGF
f
SeV
g
Rel fluor (CFP%)
Pre-bleach
Post-bleach
Mock
Insulin
EGF
SeV
PTEN + Akt1

Figure S5

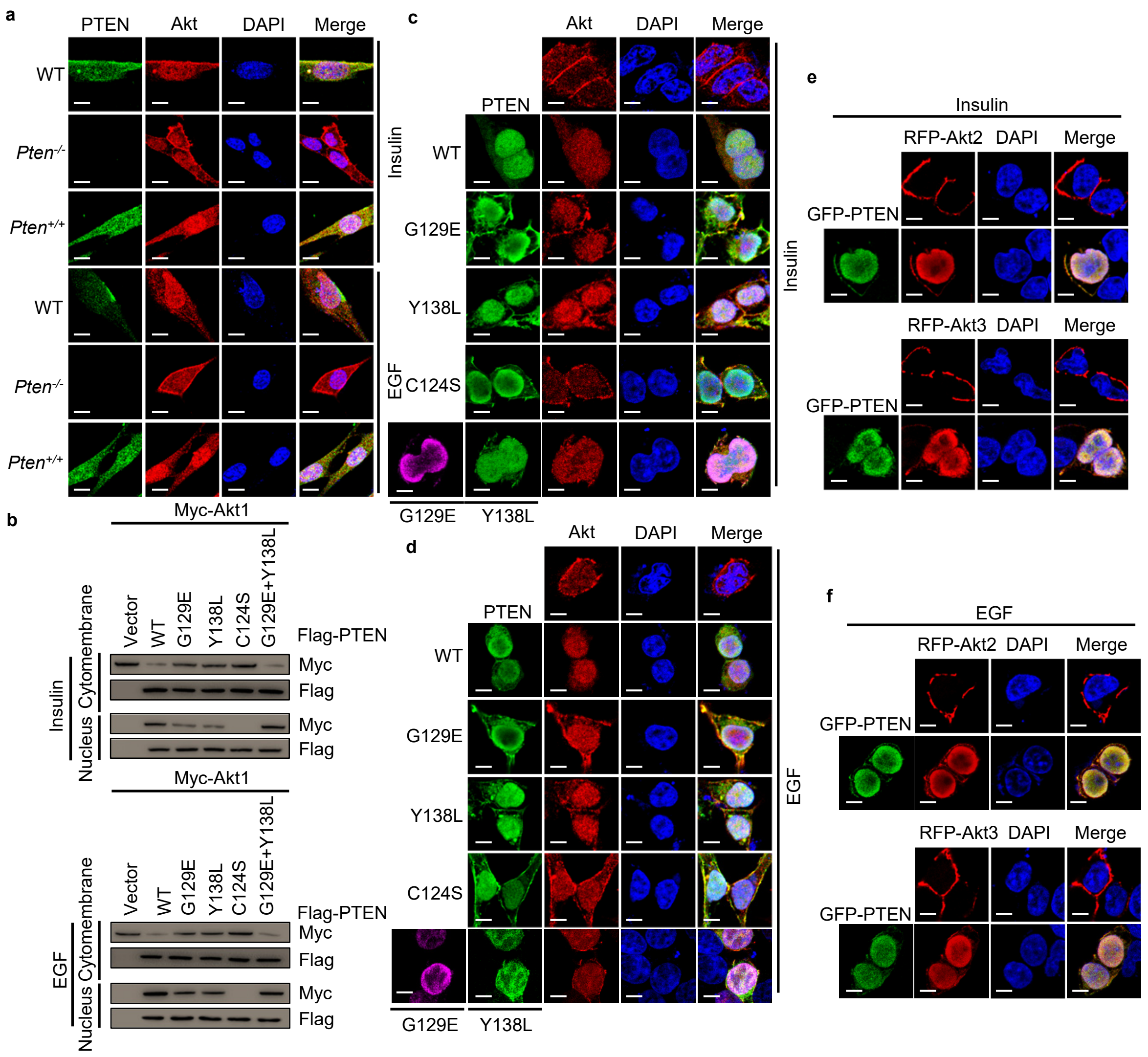
a
PTEN
Akt
DAPI
Merge
WT
Pten-/-
Pten+/+
Insulin
EGF
b
Myc-Akt1
Vector
WT
G129E
Y138L
C124S
G129E+Y138L
Flag-PTEN
Insulin
EGF
Cytomembrane
Nucleus
Myc
Flag
c
d
G129E
Y138L
C124S
e
f
RFP-Akt2
RFP-Akt3
GFP-PTEN

Figure S6

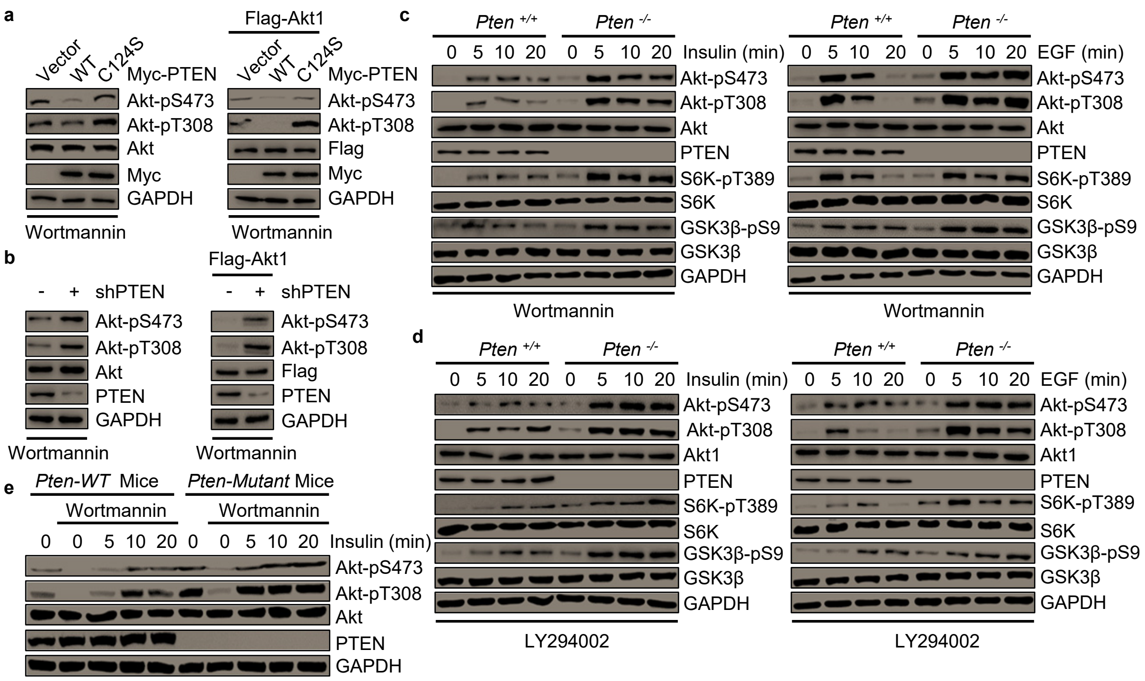

a
Flag-Akt1
Vector
WT
C124S
Myc-PTEN
Akt-pS473
Akt-pT308
Akt
Flag
Myc
GAPDH
Wortmannin
b
- + shPTEN
Akt-pS473
Akt-pT308
Akt
Flag
PTEN
GAPDH
Wortmannin
c
Pten +/+
Pten -/-
0 5 10 20 0 5 10 20 Insulin (min)
0 5 10 20 0 5 10 20 EGF (min)
Akt-pS473
Akt-pT308
Akt
PTEN
S6K-pT389
S6K
GSK3β-pS9
GSK3β
GAPDH
Wortmannin
d
Akt1
LY294002
e
Pten-WT Mice
Pten-Mutant Mice
Wortmannin
0 0 5 10 20 0 0 5 10 20 Insulin (min)
Akt-pS473
Akt-pT308
Akt
PTEN
GAPDH

Figure S7

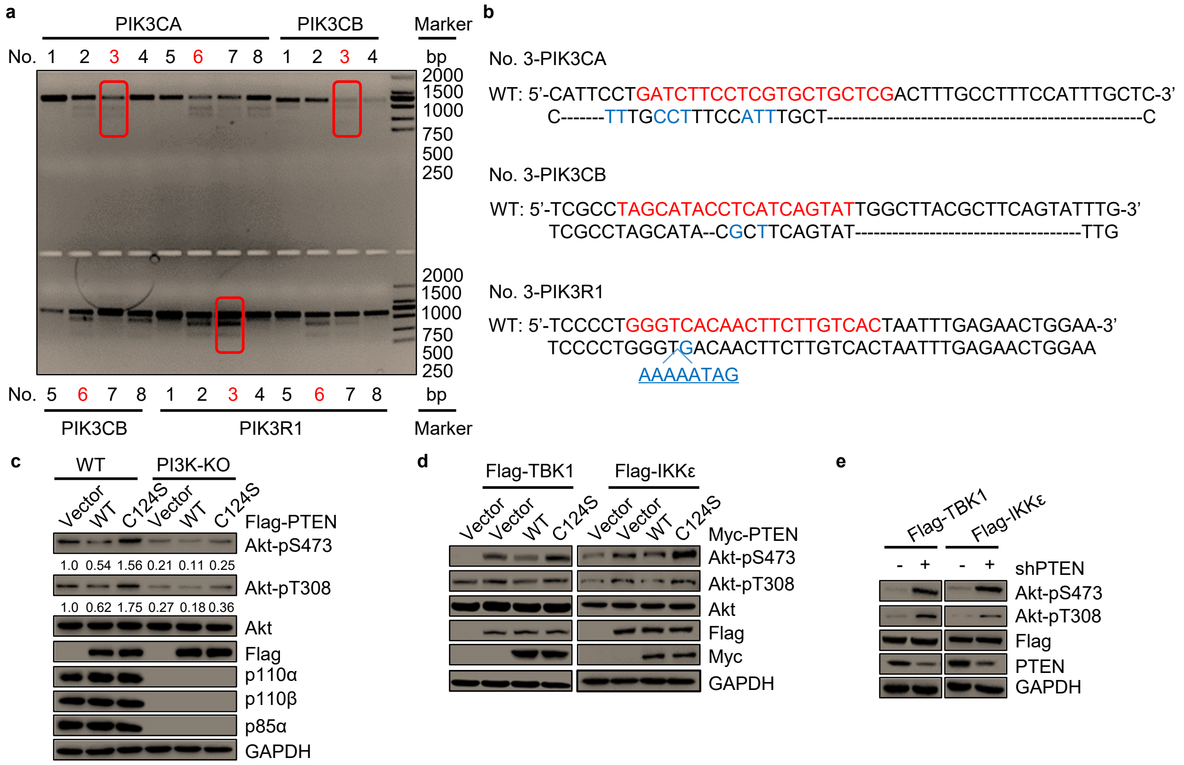
a
PIK3CA
PIK3CB
Marker
No. 1 2 3 4 5 6 7 8 1 2 3 4
bp
2000
1500
1000
750
500
250
No. 5 6 7 8 1 2 3 4 5 6 7 8
PIK3CB
PIK3R1
b
No. 3-PIK3CA
WT: 5'-CATTCCTGATCTTCCTCGTGCTGCTCGACTTTGCCTTTCCATTTGCTC-3'
C-------TTTGCCTTTCCATTTGCT--C
No. 3-PIK3CB
WT: 5'-TCGCCTAGCATACCTCATCAGTATTGGCTTACGCTTCAGTATTTG-3'
TCGCCTAGCATA--CGCTTCAGTAT-----------------------------------TTG
No. 3-PIK3R1
WT: 5'-TCCCCTGGGTCACAACTTCTTGTCACTAATTTGAGAACTGGAA-3'
TCCCCTGGGTGACAACTTCTTGTCACTAATTTGAGAACTGGAA
AAAAATAG
c
WT
PI3K-KO
Vector WT C124S Vector WT C124S
Flag-PTEN
Akt-pS473
1.0 0.54 1.56 0.21 0.11 0.25
Akt-pT308
1.0 0.62 1.75 0.27 0.18 0.36
Akt
Flag
p110α
p110β
p85α
GAPDH
d
Flag-TBK1
Flag-IKKε
Vector Vector WT C124S Vector Vector WT C124S
Myc-PTEN
Akt-pS473
Akt-pT308
Akt
Flag
Myc
GAPDH
e
Flag-TBK1
Flag-IKKε
- + - +
shPTEN
Akt-pS473
Akt-pT308
Flag
PTEN
GAPDH

Figure S8

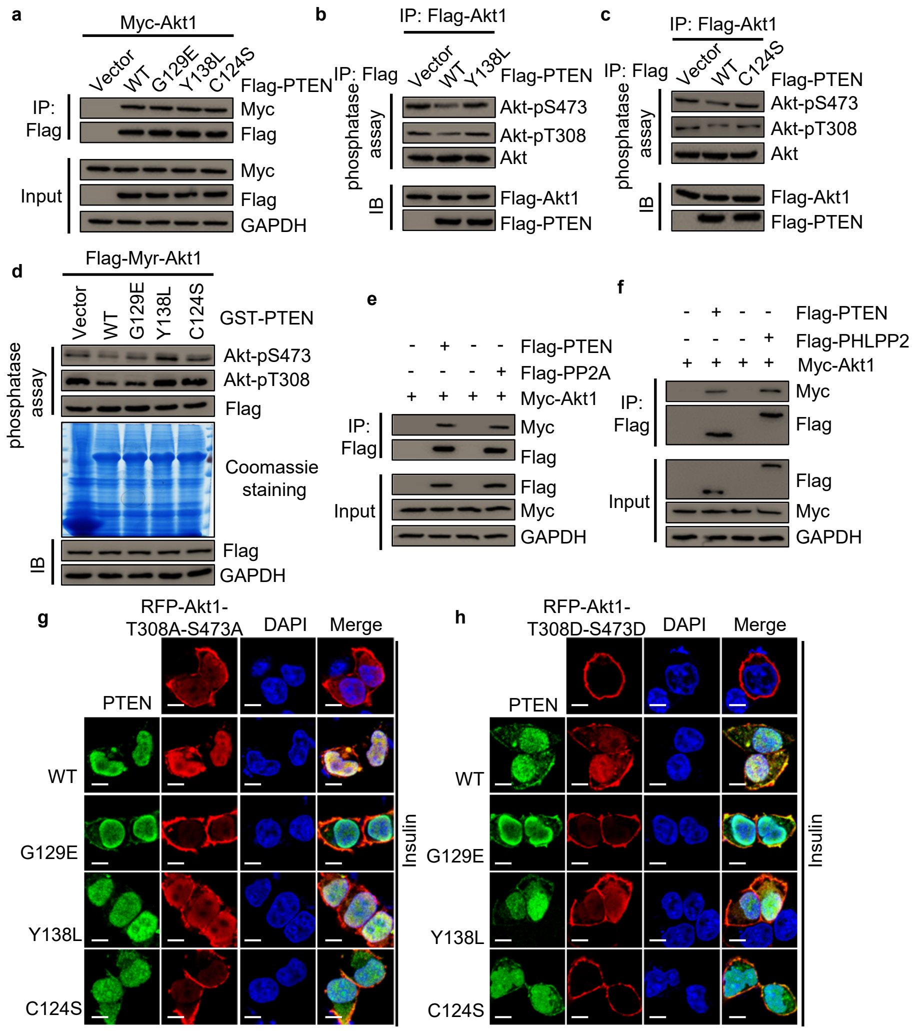

a
Myc-Akt1
Vector
WT
G129E
Y138L
C124S
Flag-PTEN
IP: Flag
Myc
Flag
Input
Myc
Flag
GAPDH
b
IP: Flag-Akt1
IP: Flag
Vector
WT
Y138L
Flag-PTEN
phosphatase assay
Akt-pS473
Akt-pT308
Akt
IB
Flag-Akt1
Flag-PTEN
c
IP: Flag-Akt1
IP: Flag
Vector
WT
C124S
Flag-PTEN
phosphatase assay
Akt-pS473
Akt-pT308
Akt
IB
Flag-Akt1
Flag-PTEN
d
Flag-Myr-Akt1
Vector
WT
G129E
Y138L
C124S
GST-PTEN
phosphatase assay
Akt-pS473
Akt-pT308
Flag
Coomassie staining
IB
Flag
GAPDH
e
Flag-PTEN
Flag-PP2A
Myc-Akt1
IP: Flag
Myc
Flag
Input
Flag
Myc
GAPDH
f
Flag-PTEN
Flag-PHLPP2
Myc-Akt1
IP: Flag
Myc
Flag
Input
Flag
Myc
GAPDH
g
RFP-Akt1-T308A-S473A
DAPI
Merge
PTEN
WT
G129E
Y138L
C124S
Insulin
h
RFP-Akt1-T308D-S473D
DAPI
Merge
PTEN
WT
G129E
Y138L
C124S
Insulin

Figure S9

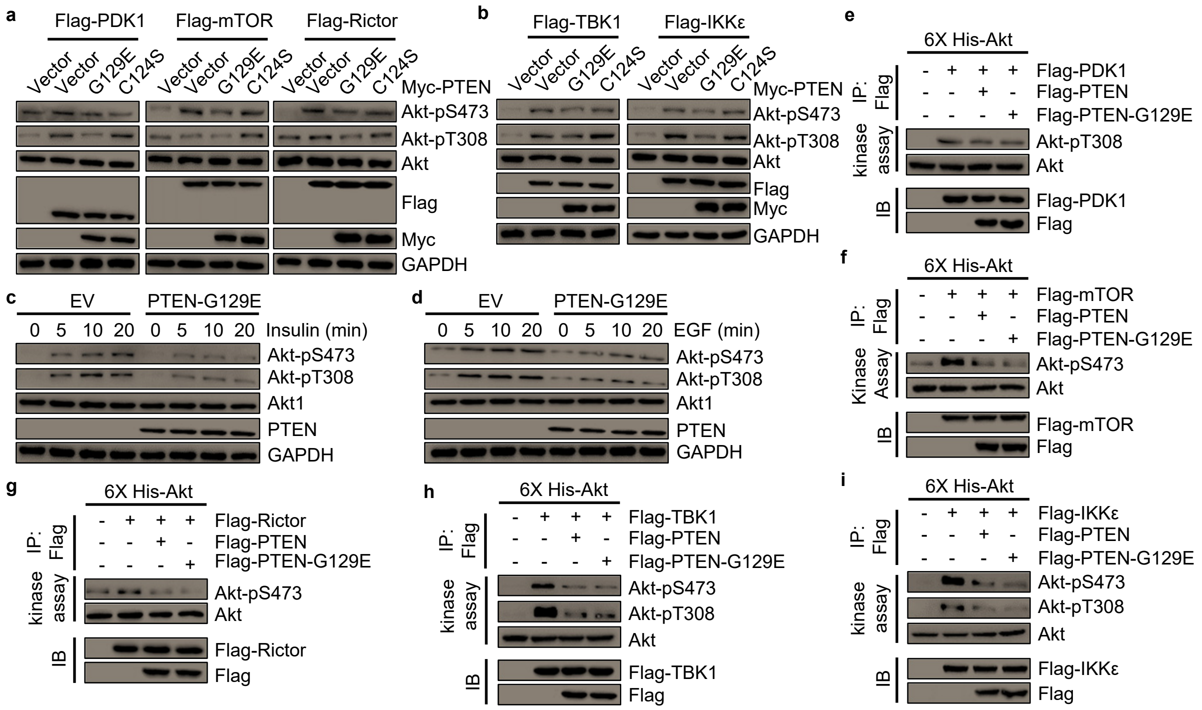

a
Flag-PDK1
Flag-mTOR
Flag-Rictor
Vector
Vector
G129E
C124S
Myc-PTEN
Akt-pS473
Akt-pT308
Akt
Flag
Myc
GAPDH
b
Flag-TBK1
Flag-IKKε
c
EV
PTEN-G129E
0 5 10 20 0 5 10 20 Insulin (min)
Akt1
PTEN
d
EGF (min)
e
6X His-Akt
IP: Flag
Flag-PDK1
Flag-PTEN
Flag-PTEN-G129E
kinase assay
IB
f
Flag-mTOR
Kinase Assay
g
Flag-Rictor
h
Flag-TBK1
i
Flag-IKKε

Figure S10

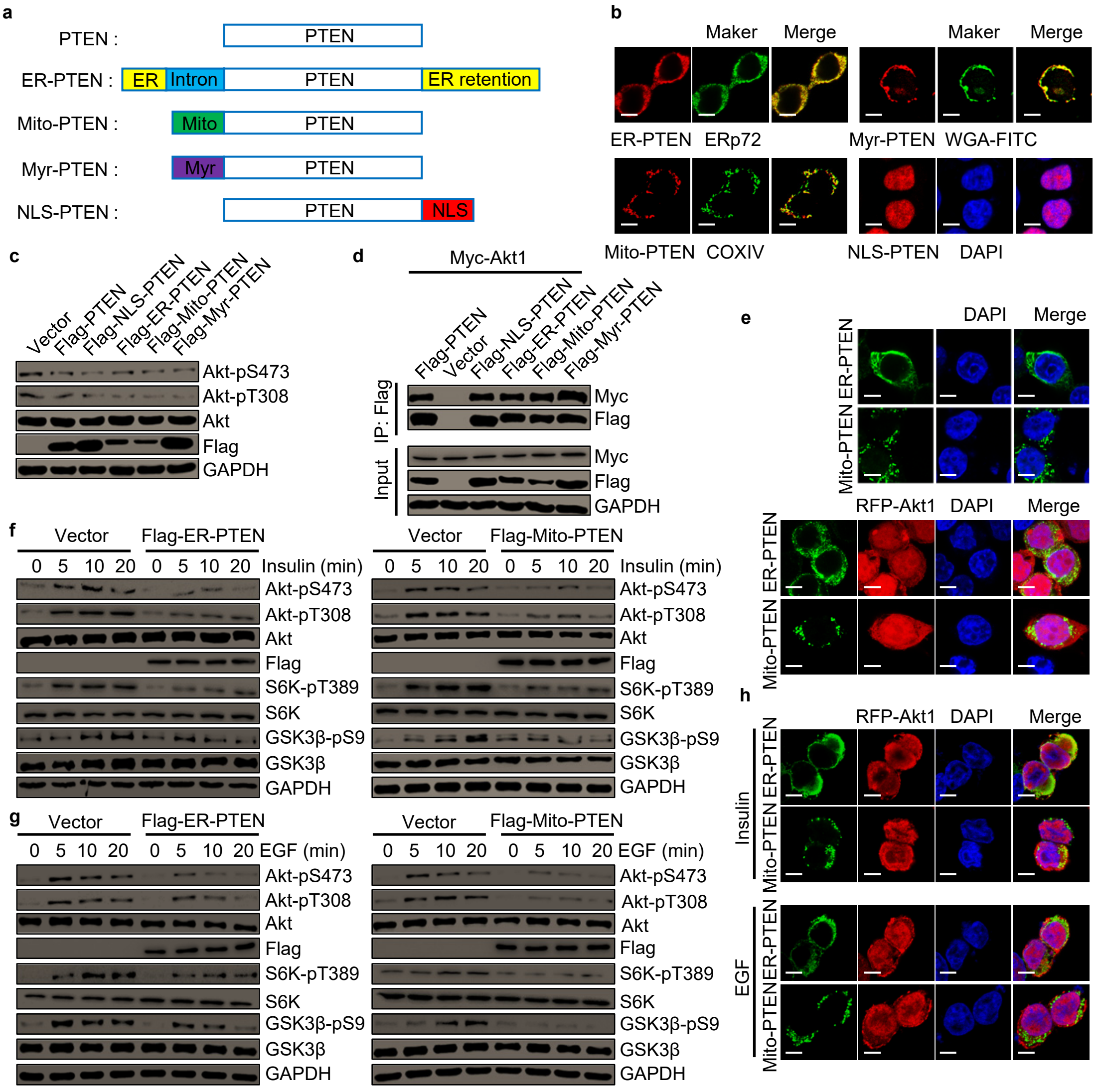
a
PTEN : PTEN
ER-PTEN : ER Intron PTEN ER retention
Mito-PTEN : Mito PTEN
Myr-PTEN : Myr PTEN
NLS-PTEN : PTEN NLS
b
Maker Merge Maker Merge
ER-PTEN ERp72 Myr-PTEN WGA-FITC
Mito-PTEN COXIV NLS-PTEN DAPI
c
Vector Flag-PTEN Flag-NLS-PTEN Flag-ER-PTEN Flag-Mito-PTEN Flag-Myr-PTEN
Akt-pS473
Akt-pT308
Akt
Flag
GAPDH
d
Myc-Akt1
Flag-PTEN Vector Flag-NLS-PTEN Flag-ER-PTEN Flag-Mito-PTEN Flag-Myr-PTEN
IP: Flag
Input
Myc
Flag
GAPDH
e
DAPI Merge
ER-PTEN
Mito-PTEN
RFP-Akt1 DAPI Merge
f
Vector Flag-ER-PTEN Vector Flag-Mito-PTEN
0 5 10 20 0 5 10 20 Insulin (min)
Akt-pS473
Akt-pT308
Akt
Flag
S6K-pT389
S6K
GSK3β-pS9
GSK3β
GAPDH
g
Vector Flag-ER-PTEN Vector Flag-Mito-PTEN
0 5 10 20 0 5 10 20 EGF (min)
h
RFP-Akt1 DAPI Merge
Insulin
EGF

Figure S11

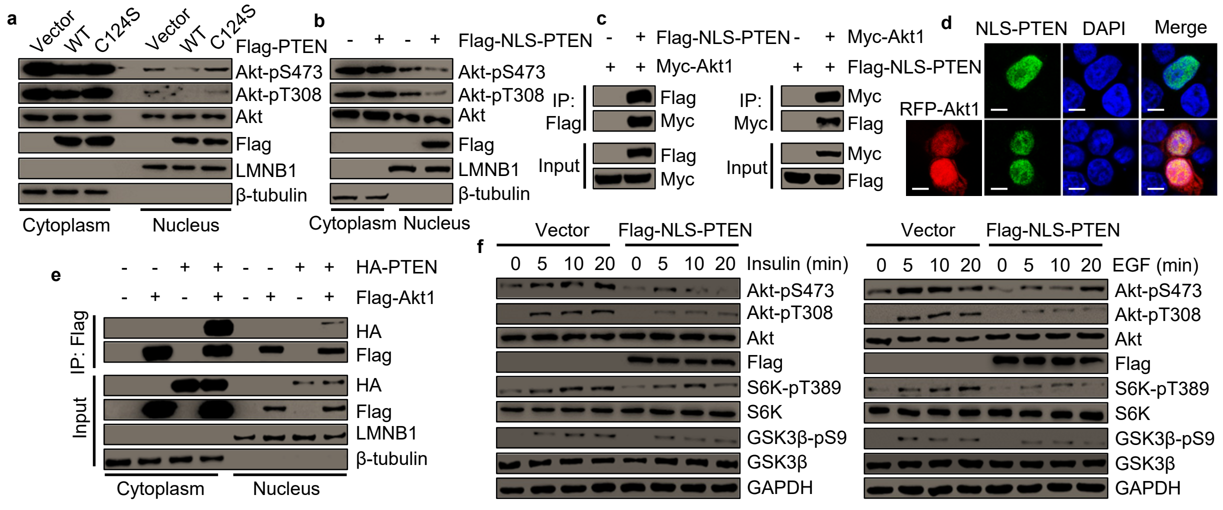
a
Vector
WT
C124S
Vector
WT
C124S
Flag-PTEN
Akt-pS473
Akt-pT308
Akt
Flag
LMNB1
β-tubulin
Cytoplasm
Nucleus
b
Flag-NLS-PTEN
Akt-pS473
Akt-pT308
Akt
Flag
LMNB1
β-tubulin
Cytoplasm Nucleus
c
Flag-NLS-PTEN
Myc-Akt1
IP: Flag
Flag
Myc
Input
Myc-Akt1
Flag-NLS-PTEN
IP: Myc
Input
d
NLS-PTEN
DAPI
Merge
RFP-Akt1
e
HA-PTEN
Flag-Akt1
IP: Flag
HA
Flag
Input
LMNB1
β-tubulin
Cytoplasm
Nucleus
f
Vector
Flag-NLS-PTEN
0 5 10 20 0 5 10 20 Insulin (min)
0 5 10 20 0 5 10 20 EGF (min)
Akt-pS473
Akt-pT308
Akt
Flag
S6K-pT389
S6K
GSK3β-pS9
GSK3β
GAPDH

Figure S12

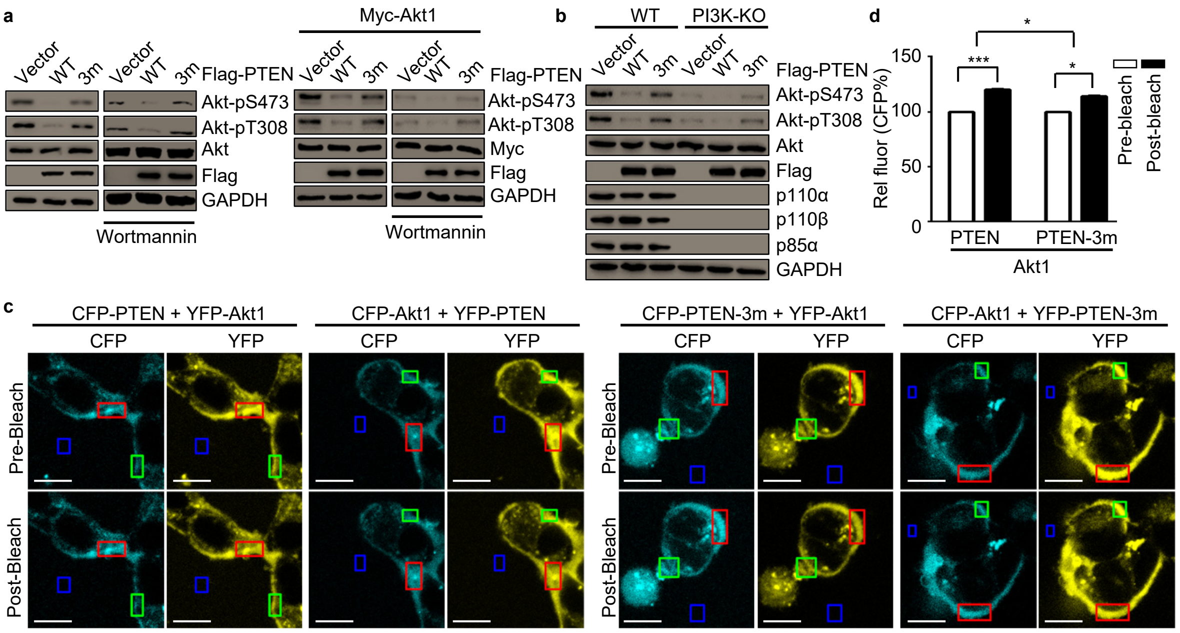
a
Myc-Akt1
Vector
WT
3m
Flag-PTEN
Akt-pS473
Akt-pT308
Akt
Flag
GAPDH
Wortmannin
Myc
b
WT
PI3K-KO
p110α
p110β
p85α
d
Rel fluor (CFP%)
150
100
50
0
***
*
Pre-bleach
Post-bleach
PTEN
PTEN-3m
Akt1
c
CFP-PTEN + YFP-Akt1
CFP-Akt1 + YFP-PTEN
CFP-PTEN-3m + YFP-Akt1
CFP-Akt1 + YFP-PTEN-3m
CFP
YFP
Pre-Bleach
Post-Bleach

Figure S13

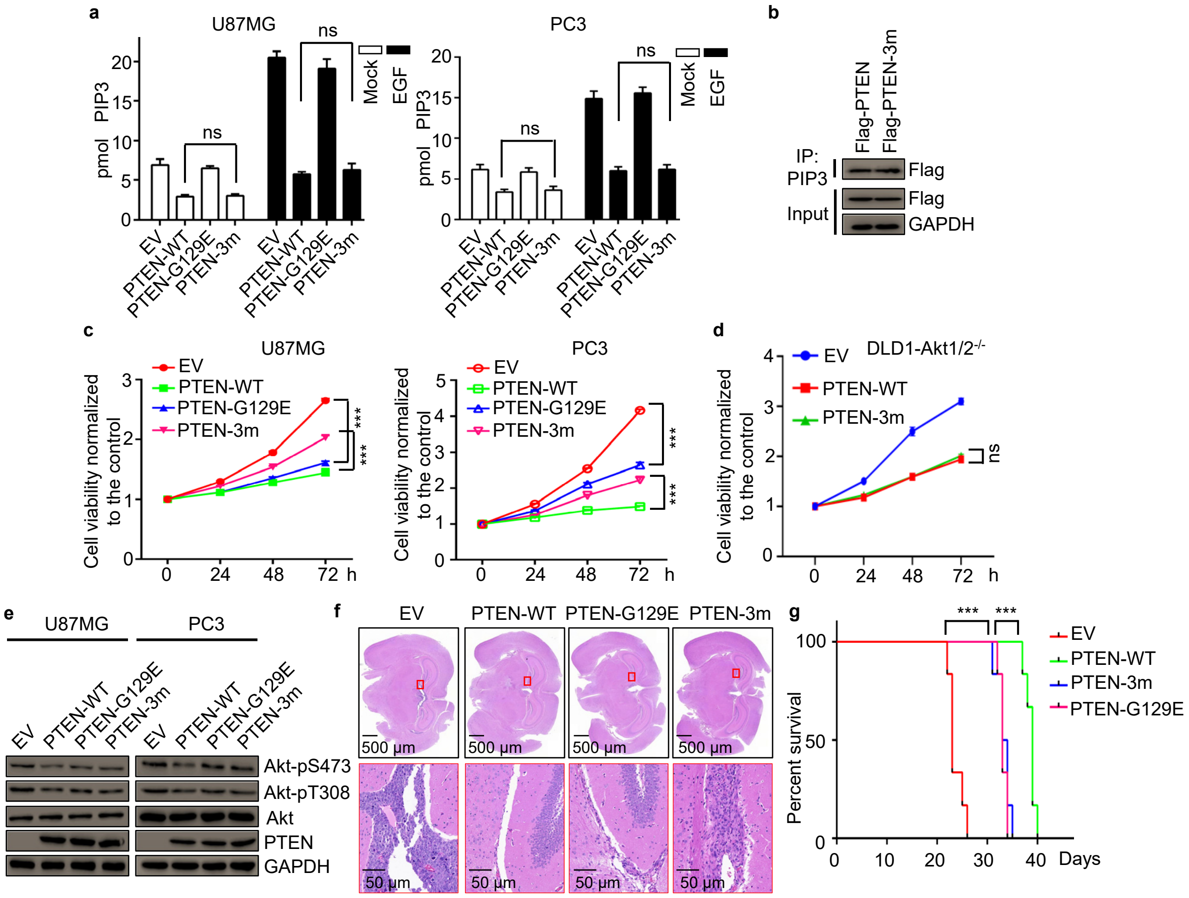

a
U87MG
PC3
pmol PIP3
ns
Mock
EGF
EV
PTEN-WT
PTEN-G129E
PTEN-3m
b
Flag-PTEN
Flag-PTEN-3m
IP: PIP3
Input
Flag
GAPDH
c
Cell viability normalized to the control
***
d
DLD1-Akt1/2-/-
e
Akt-pS473
Akt-pT308
Akt
PTEN
f
500 µm
50 µm
g
Percent survival
Days
